# Proposing a material selection indicator for the design of extended lifespan products

**DOI:** 10.1038/s41598-025-21186-0

**Published:** 2025-10-24

**Authors:** Marco Granados-Sarmiento, Jenaan Tarabein-Omairi, Humberto Gomez, Jaime A. Mesa

**Affiliations:** 1 Department of Mechanical Engineering, Universidad del Norte, Barranquilla, Colombia; 2 GIMYP Research Unit, Department of Mechanical Engineering, Universidad del Norte, Barranquilla, 081001 Colombia

**Keywords:** Durability, Material selection, Extended lifespan, Circular economy, Sustainable design, Energy infrastructure, Mechanical engineering, Environmental sciences, Materials for devices

## Abstract

Material durability has emerged as a key factor influencing product longevity, safety, and cost-effectiveness across various sectors, including the construction, automotive, and electronics sectors. With the growing emphasis on sustainability, there is an increasing demand for materials that not only exhibit high performance but also align with circular economy practices such as reuse, repair, remanufacturing, and repurpose. The challenge lies in the existing material selection frameworks, which frequently fail to comprehensively integrate multiple aspects of durability with environmental impact. Most current indicators predominantly focus on ensuring material durability over time without sufficiently considering the complete lifecycle environmental footprint. To address these challenges, this study introduces the Specific Durability Performance, a quantitative approach that merges mechanical, thermal, and chemical durability with a carbon footprint into a singular value ranging from 0.0 to 1.0. The methodology involves calculating key parameters for each material, such as tensile strength, fatigue resistance, service temperature range, flammability, and chemical resistance, which are weighted according to the criteria that assess the specific use requirements of the product. Each dimension of durability was benchmarked against an “ideal” reference material, and the carbon footprint was also benchmarked. These results were then synthesized into a unique score designed to facilitate material selection by balancing performance and sustainability. This approach was applied to two case studies with distinct design requirements, demonstrating the usefulness of the proposed indicator in identifying materials that offer robust structural performance and reduced environmental impact.

**Supplementary Information:**

The online version contains supplementary material available at 10.1038/s41598-025-21186-0.

## Introduction

Material durability is a critical factor in the selection of materials for a wide range of industrial applications, directly affecting the product lifespan, safety, and cost-effectiveness. Durability refers to the capacity of a material to withstand the conditions of its environment and use without significant degradation over time^1^. This attribute is critical in industries such as construction, automotive, and electronics, where the ability of materials to endure can determine the overall product performance and competitiveness.

The mechanical, chemical, and thermal durability of materials determines their longevity and performance in various applications. Mechanical durability is often evaluated based on properties such as tensile strength, impact resistance, and fatigue life, which are essential for ensuring the structural integrity of materials under various loads and conditions^2^. Chemical durability is related to a material’s resistance to degradation by chemicals, including corrosion and other chemical reactions that could compromise the integrity of the material over time^3^. Thermal durability assesses a material’s ability to withstand temperature fluctuations and thermal stress without significant degradation, as it degrades the process by which heat or elevated temperatures act on a material, product, or assembly, resulting in the degradation of its properties^4–6^. As industries face pressure to enhance sustainability, the need for materials with high durability to reduce waste and improve life-cycle efficiency has become more critical, making material selection a fundamental process in modern design and engineering^7^. Currently, effective material selection outlines the lifecycle of products, especially in supporting sustainable practices such as reuse, repair, remanufacturing, refurbishment, and repurpose^8^.

The selection of materials with high durability against mechanical, chemical, and thermal stresses allows engineering designers to enhance product longevity, minimize waste, and enable secondary applications. Choosing materials that endure multiple life cycles supports the transition to a circular economy, enabling components to be efficiently repaired or repurposed, ultimately conserving resources, generating social and economic value, and minimizing environmental impacts^9-11^. Incorporating carbon footprint into the material selection process is currently a priority in the context of global efforts to reduce greenhouse gas emissions and combat climate change. The carbon footprint of a material encompasses all emissions associated with its production, transportation, usage, and disposal, or, in general, all the emissions emitted over the entire life cycle of a product^12^.

Most existing material selection indicators follow a framework that focuses on ensuring material durability over time^13–15^. This is particularly crucial for materials used in electrical and electronic products, which have seen a significant decline in lifespan owning to rapid technological advancements and changing consumer demands^16^. Other indicators emphasize measuring the degradation of materials throughout their lifecycle to better understand the stage of circularity at which a product exists^17,18^. The design for durability aligns with essential strategies in the circular economy, helping maintain product value throughout its lifecycle within a defined technological period. However, there is a need to reevaluate and realign durability criteria to address new requirements, material properties, and business models, such as the product service approach, which involves more durable and reliable products.

To contribute to this research direction, this study introduces the Specific Durability Performance (SDP), designed to facilitate material selection during the product design stage by incorporating the product’s use context and its lifecycle environmental impact. In practice, this means the SDP indicator is intended for use during the embodiment design phases, when designers are screening and selecting materials to meet product requirements. Applying SDP at this stage ensures durability and sustainability criteria guide the material selection before final design decisions are locked in. Two key features of the SDP are: (i) the integration of chemical, thermal, and mechanical durability with environmental impact into a single calculation. (ii) Additionally, it utilizes contextual questions about the product to determine which properties require greater emphasis during the design process, further enhancing decision-making across specific material options or entire families of materials.

The proposed indicator seeks to assist designers in choosing materials that achieve an optimal balance between performance and minimal ecological harm. In a circular economy, designing products for longevity, through durability, ease of repair, and adaptability, is crucial to keep resources in use and minimize waste. To operate circular economy principles in early design the SDP proposes a composite metric to guide material selection for extended lifespan products. Durable, long-lasting materials ensure that products can be used longer and even given second or third lives, rather than being discarded, directly contributing to circular economy objectives. The SDP indicator ensures a balanced approach, encouraging selection of materials that maximize durability while minimizing carbon footprint, thereby addressing both longevity and sustainability in one metric. While much of the circular economy discussion has focused on recycling and end-of-life recovery, strategies that extend product life and yield even higher circularity and value retention^19^.

 The structure of this article is organized as follows: Sect. 2 presents a comprehensive literature review, focusing on current indicators pertinent to material selection and those within the circular economy framework. Section 3 introduces the proposed indicator and elaborates on its parameters. Section 4 demonstrates the application of the SDP indicator through two case studies. Section 5 discusses the findings and interprets the results. Finally, Sect. 6 provides a summary of the conclusions and offers recommendations based on the research outcomes.

## Literature review

**Fig. 1:**
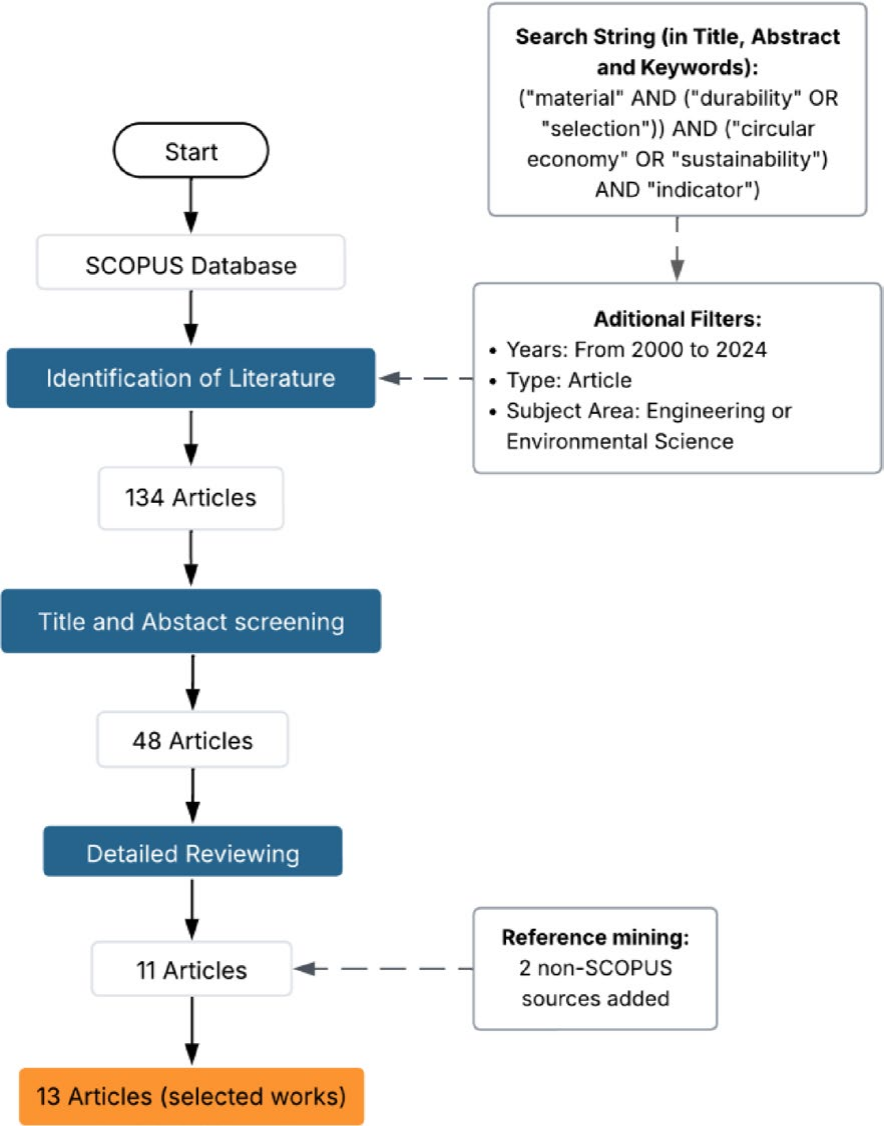
Summary of literature search and selection of relevant works.

This section reviews the existing research on durability indicators, particularly those that support material selection for long-lasting products in engineering contexts. Studies have examined the mechanical, thermal, and chemical aspects of durability, as well as the carbon footprint, which are essential for comprehensive assessments and are aligned with the growing emphasis on sustainability and efficient resource utilization. The review focused on SCOPUS-indexed articles published between 2000 and 2024 in engineering and environmental science journals. Initially, abstracts were screened to assess relevance based on criteria such as scope, methodology, and results, with particular attention to material durability, material selection, and circular economy strategies. This was followed by an in-depth review of the most relevant works to ensure a direct connection with the research focus. The complete methodology applied in the literature review process is visually summarized in Fig. 1, which includes the search strategy, filtering steps, and final inclusion of 13 selected studies.

### **Material durability evolution**

Material durability started gaining relevance in the late 20th century, when life cycle design (LCD) emerged, representing a major turning point in sustainability practices. Traditionally, product design and process design have been treated separately, but LCD integrates them into a single function to reduce the added environmental impact associated with product systems more effectively^20^. This emphasizes the use of durable materials to reduce waste and minimize the need for frequent replacements^21^. Aligning product and process design allowed LCD to foster a more integrated sustainability framework, emphasizing durability, efficiency, and environmental management across the entire product life cycle^22^.

By the early 2000 s, material durability was viewed not only in terms of physical lifespan, but also in relation to environmental sustainability^23^. Researchers have begun to explore the broader impact of material longevity on resource consumption, waste management, and energy use^24,25^. Durable materials have become central to reducing environmental impacts, considering factors such as recyclability, disassembly, and potential circular economic benefits. This growing focus on renewable resources and waste minimization underscores the importance of choosing materials that could be reused or recycled after a product’s functional life.

Later, the discussion about low-impact resource selection was expanded to include more detailed considerations of material durability. Designers now assess the entire lifecycle of a material, from extraction to disposal, factoring in its potential for recovery, reuse, and biodegradation^26^. The Cradle-to-Cradle philosophy promotes materials that can continually cycle in closed loops, ensuring that they do not contribute to environmental degradation even after their functional life ends^27^.

Today, material durability continues to evolve with an increasing emphasis on designing products for longevity, recyclability, and minimal environmental impact^28,29^. Current research focuses on integrating and combining Life Cycle Assessment (LCA) techniques with innovative methodologies to identify gaps and opportunities to optimize resource use and minimize environmental impacts throughout all stages of the product life cycle^30,31^.

### Material durability indicators and frameworks

Based on the literature review, previous research related to material durability indicators and frameworks is presented and summarized in Table 1. The authors, name of the indicator, aim, and three key dimensions used for comparative analysis: the product life cycle stage(s) it addresses, the material family considered, and whether it is oriented toward product design. The first of these dimensions, helps clarify the specific context in which each indicator operates. These stages are based on the general system boundaries described in LCA (ISO 14040/14044) and have been expanded to reflect principles of the circular economy. Rather than being interpreted as a strict sequence, they are treated as distinct analytical categories that highlight how each indicator contributes to product durability and circularity. They include:

**Table 1:** Key material durability indicators and frameworks for material selection.

Author	Indicator	Description	Product Life Cycle Stage	Material family	Product Design-oriented?
^16^	Durability Index Framework	Measuring and indexing the durability of electrical and electronic equipment to promote a circular economy.	Manufacturing, Use	Metals, Ceramics	No
^32^	MEM: Material Efficiency Metric	Determine how ME strategies can affect extraction rates of virgin material and how waste creation can be reduced.	Extraction, Manufacturing, Recycling	All	No
^17^	Functional Circularity indicator	Indicates a formula for the degradation of a product over time.	Re-use, Recycling, Use	All	No
	FHL: Functional Half Life	States the time a product has lost a fraction of its functionality.	Recover, Use	All	No
^18^	UOR: In-use Occupation Ratio	The performance of the entire occupation for the use of the material	Manufacturing, Recover, Remanufacturing, Use	Raw Materials	No
	FRS: Final Retention in Society	The remaining percentage of the primary raw material after a period of product cycle.	Recycling, Use	Raw Materials	No
^33^	PCI: Product Circularity Indicator	Use of material considering different manufacturing steps and the associated material losses are accounted for as waste or recycled material.	Manufacturing, Recycling, Use	All	No
^19^	MDI: Material Durability Indicator	Durability and environmental footprint for material selection in a Circular Economy context.	Manufacturing, Use	All	Yes
^34^	Displacement rate	CE performance through the use of recycled and re-used materials as substitutes or complements of primary materials.	Re-use, Recycling	All	No
^35^	GRI: Global Resource Indicator	Performance of resources considering recyclability and criticality through multi-criteria analysis.	Material Extraction, Recycling, Use	All	No
^36^	VRE: Valued-based Resource Efficiency	Resource efficiency and CE performance in terms of market value considering scarcity, competition, and social and environmental externalities.	Recover, Recycling	All	No
^37^	PLCM: Product Level Circularity Metric	Economic value and circularity of product parts and value chain activities considering materials recirculation.	Remanufacturing, Recycling, Use	All	Yes
^38^	Eco-costs	Ratio between LCA impact and economic value.	Extraction, Use	No Materials	No
^39^	MCI: Material Circularity Indicator	Use of virgin material and resulting waste sent to landfill or energy recovery.	Manufacturing, Recycling, Use	All	Yes
^27^	Material Reutilization part – Cradle to Cradle	Degree of material re-use in the development of products.	Re-use, Recovery	All	No

- Extraction: obtaining raw materials, where durability considerations can reduce resource consumption and reliance on critical inputs.
- Manufacturing: converting materials into products, with attention to minimizing waste and avoiding early degradation.
- Use: the period of active product operation, where long-term performance and reliability are essential.
- Re-use and Remanufacturing: extending the functional life of products either through direct reuse or through repair and restoration.
- Recycling: recovering materials to be reintroduced into production, reducing the need for virgin inputs.
- Recovery: capturing remaining value (often as energy) when other circular strategies are no longer viable.

Attributing these stages to each indicator enables a systematic mapping of how durability is addressed throughout the product life cycle. This approach not only identifies phases that remain less developed in the literature, such as Use and Remanufacturing, but also underscores the importance of examining the entire spectrum, from Extraction and Manufacturing to Recycling and Recovery, to capture the full circularity potential of materials. The additional dimensions of material family and design orientation further clarify whether an indicator is tailored to specific material classes or can be applied broadly, and whether it supports decision-making at the product development stage. The holistic consideration of these three dimensions allows for a more balanced comparison across existing frameworks and highlight opportunities to strengthen durability assessment at multiple stages of the life cycle, rather than privileging any single phase.

Based on Table 1, several limitations have been categorized into key thematic areas to elucidate where existing tools may inadequately support sustainable design practices.

- Orientation gap toward design processes: A significant number of existing indicators focus on attributes related to material extraction, manufacturing, and recycling stages, rather than providing guidance for designers during the product development phase. This deficiency highlights the necessity for designer-oriented tools that integrate sustainability considerations from the inception of the design process.
- Lack of metric comprehensiveness: Some indicators assess durability and environmental impact but fail to consolidate mechanical, chemical, and thermal durability into a singular, comprehensive metric.
- Life-cycle stage specificity: Numerous indicators emphasize specific phases rather than encompassing the entire product lifecycle.

## Proposing the specific durability performance approach

The SDP indicator integrates four primary factors, namely chemical durability, mechanical durability, thermal durability, and environmental performance. Each factor comprises a well-researched subset of engineering parameters or properties. The SDP can be interpreted as a balance between the three durabilities and the environmental impacts associated with the material lifecycle (primary production, processing, and recycling). Consequently, the maximum value of the SDP corresponds to the material exposing optimal performance to durability and environmental burden, regarding the best possible material that could be chosen among the evaluated materials. Thus, the SDP varies from 0.0 to 1.0.

The SDP employs an idealized reference material, termed “Material X,” to benchmark candidate materials against optimal performance across mechanical, thermal, chemical, and environmental criteria, yielding a normalized score between 0.0 and 1.0. This approach facilitates a standardized comparison by defining an upper performance limit, acknowledging that real materials often involve trade-offs between categories (e.g., high mechanical durability may correlate with higher environmental impact). The normalization and weighting of these categories, determined through context-specific questions (Sect. 3.2), aim to balance these trade-offs by prioritizing properties relevant to the product’s application, as demonstrated in the case studies. While the use of an ideal material may suggest that excellent performance in one category could offset poorer performance in another, the SDP mitigates this by assigning weights based on application-specific requirements, ensuring that critical properties are prioritized. An alternative approach focusing on minimum industry requirements for pre-selection was considered but deemed less suitable for this study, as it risks limiting the exploration of materials that exceed baseline standards, which is crucial for advancing sustainable design. Future research could integrate minimum thresholds alongside the SDP to address industry specific constraints while maintaining the focus on optimizing durability and environmental performance.

It is important to note that the SDP score is a dimensionless index and not a direct measure of service life. Instead, a higher SDP suggests that a material possesses durability attributes that can support a longer product lifespan, whereas a lower SDP indicates that durability limitations may lead to a shorter useful life. For instance, the SDP integrates durability factors (mechanical, chemical, thermal) with embodied carbon footprint, a material scoring high in SDP would be expected to maintain its integrity for a longer period under equivalent conditions than a material scoring low. It should be emphasized that the impacts of maintenance or use-phase activities are not included in the SDP calculation. This approach is appropriate for early-stage design decisions, especially for products, like the case studies in this work. Users can therefore use the SDP as an initial guide to material longevity and sustainability, and later incorporate detailed use-phase considerations.

The SDP indicator is primarily intended for engineers, industrial designers, and sustainability professionals involved in selecting materials for durable, eco-friendly products aligned with circular economy principles. It is also valuable for academics and researchers advancing eco-design and durability metrics, providing a standardized framework for evaluating material performance in sustainable engineering applications. The SDP supports decision-making in contexts where both durability and sustainability are critical, such as infrastructure, consumer goods, and specialized equipment, offering a clear and quantifiable metric,

The SDP was developed and initially validated through two case studies in diverse engineering design contexts, which will be detailed in a later section. These case studies assessed material options based on the SDP’s four factors, confirming its ability to differentiate materials with varying durability and environmental profiles. While direct feasibility testing with exemplary users was not conducted in this study, the case studies provided a robust foundation for evaluating the SDP’s applicability.

### SDP calculation methodology

**Fig. 2:**
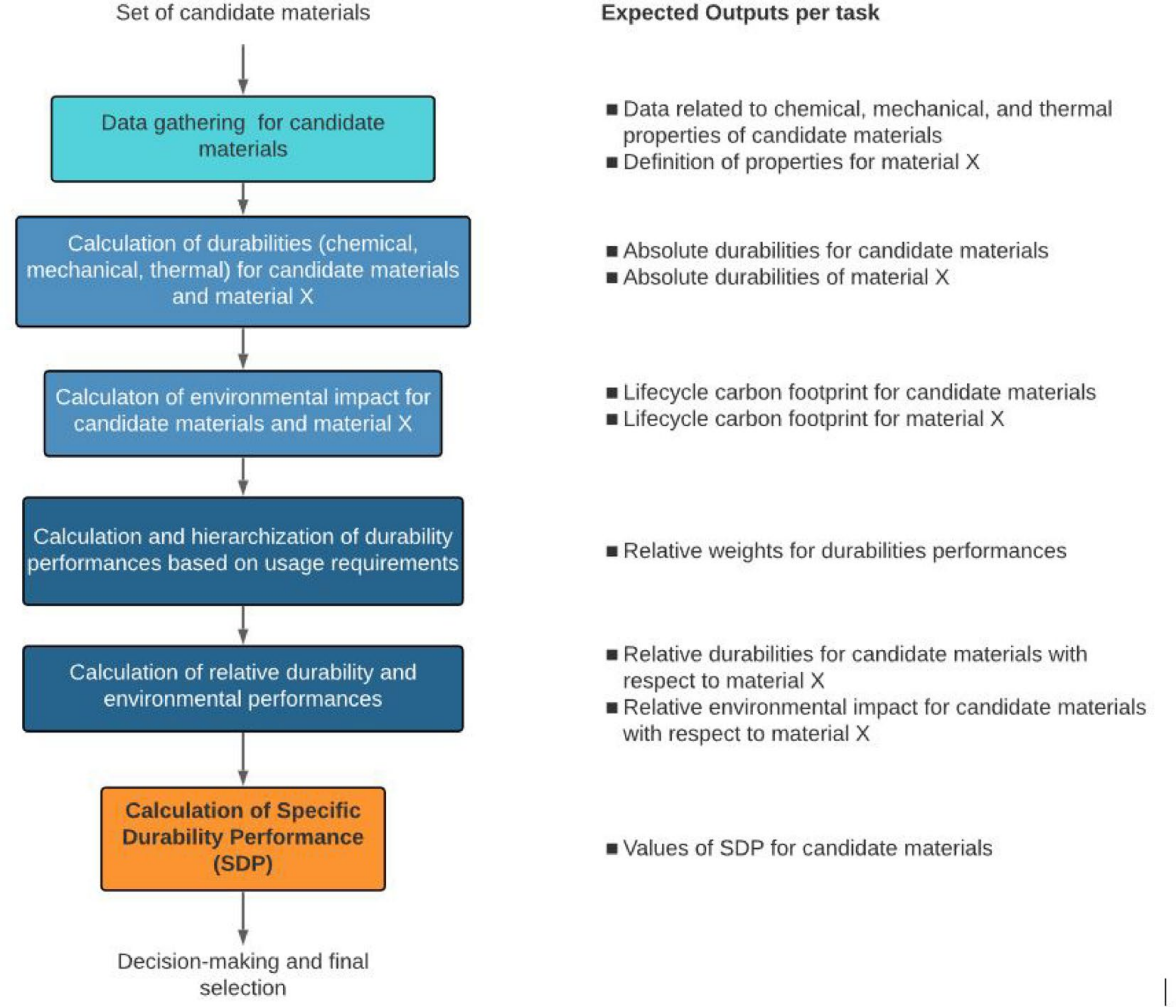
Flowchart to calculate the SDP indicator.

This section presents a comprehensive breakdown of each individual factor contributing to the SDP, providing a detailed explanation of its purpose, calculation methodology, and specific steps involved in its evaluation. Figure 2 summarizes the main steps to determine the SDP, while Eqs. 1–8 describe the calculation of the SDP. Each factor and its calculation parameters presented in the equations are then described in detail. In addition, Eqs. 3 and 8 could have subscripts *i* or X, depending on whether they refer to the candidate material or the reference material X, respectively. This distinction is necessary for their correct use in Eqs. 2 and 7:

#### Overall SDP formula

The SDP score is defined by the harmonic mean of the Durability Performance ($\:{D}_{p}$) and the Environmental Performance ($\:{E}_{p}$​):

1 $$\:SDP=2{D}_{p}{E}_{p}/({D}_{p}+{E}_{p})$$

This formula yields a value between 0 and 1, where 1 represents a material that matches the ideal reference (X-material) in both durability and environmental criteria. The sensitivity analysis of the SDP equation is found in Appendix B.

#### Durability performance

The durability performance $\:{(D}_{p})\:$of a material is expressed as a ratio between the durability of the material $\:{(D}_{i})\:$ and the durability of an ideal reference material $\:\left({D}_{x}\right):$

2 $$\:{D}_{p}={D}_{i}/{D}_{x}$$

Both $\:{D}_{i}\:$and $\:{D}_{x}\:$are calculated using the same expression, but with values corresponding to either the candidate or the ideal material. The individual durability score is obtained by weighing the mechanical, thermal, and chemical durability values:

3 $$\:{D}_{i}={M}_{D}^{{w}_{m}}\:\cdot\:{T}_{D}^{{w}_{t}}\:\cdot\:{C}_{D}^{{w}_{c}}$$

Where:

- $$\:{M}_{D}: \: {\rm Mechanical\: durability\: factor}$$

- $${T}_{D}:\text{T}\text{h}\text{e}\text{r}\text{m}\text{a}\text{l}\:\text{d}\text{u}\text{r}\text{a}\text{b}\text{i}\text{l}\text{i}\text{t}\text{y}\:\text{f}\text{a}\text{c}\text{t}\text{o}\text{r}$$

- $$\:{C}_{D}:\text{C}\text{h}\text{e}\text{m}\text{i}\text{c}\text{a}\text{l}\:\text{d}\text{u}\text{r}\text{a}\text{b}\text{i}\text{l}\text{i}\text{t}\text{y}\:\text{f}\text{a}\text{c}\text{t}\text{o}\text{r}$$

- $\:{w}_{m}$, $\:{w}_{t}$, $\:{w}_{c}:\:$Weighting coefficients for mechanical, thermal, and chemical durability, respectively. These coefficients are obtained from the 21 evaluation questions listed later in this document and should add up to 1.0.

The mechanical durability ($\:{M}_{D}$) is calculated as the geometric mean of four mechanical properties:

4 $$\:\:{M}_{D}=\sqrt[3]{{A}_{1}\cdot\:{A}_{2}\cdot\:{A}_{3}}$$

Where:

- $${A}_{1}:\:\text{Y}\text{i}\text{e}\text{l}\text{d}\:\text{s}\text{t}\text{r}\text{e}\text{n}\text{g}\text{t}\text{h}\:\left({\sigma\:}_{y}\right)$$

- $$\:{A}_{2}:\:\text{F}\text{a}\text{t}\text{i}\text{g}\text{u}\text{e}\:\text{s}\text{t}\text{r}\text{e}\text{n}\text{g}\text{t}\text{h}\:$$ $$\:\left({S}_{f}\right)$$

- $$\:{A}_{3}:\text{Y}\text{o}\text{u}\text{n}\text{g}\:\text{m}\text{o}\text{d}\text{u}\text{l}\text{u}\text{s}\:$$ $$\:\left(E\right)$$

Thermal durability $\:\left({T}_{D}\right)\:$evaluates resistance to temperature extremes and flammability:

5 $$\:\begin{array}{c}{T}_{D}=\left({B}_{1}-{B}_{2}\right)\cdot\:{B}_{3}\end{array}$$

Where:

- $$\:{B}_{1}:\:\text{M}\text{a}\text{x}\:\text{s}\text{e}\text{r}\text{v}\text{i}\text{c}\text{e}\:\text{t}\text{e}\text{m}\text{p}\text{e}\text{r}\text{a}\text{t}\text{u}\text{r}\text{e}\:\left({T}_{max}\right)$$

- $$\:{B}_{2}:\text{M}\text{i}\text{n}\:\text{s}\text{e}\text{r}\text{v}\text{i}\text{c}\text{e}\:\text{t}\text{e}\text{m}\text{p}\text{e}\text{r}\text{a}\text{t}\text{u}\text{r}\text{e}\:\left(\:{T}_{min}\right)\:\:$$

- $$\:{B}_{3}:\:\text{F}\text{l}\text{a}\text{m}\text{m}\text{a}\text{b}\text{i}\text{l}\text{i}\text{t}\text{y}$$

Chemical durability $\:\left({C}_{D}\right)\:$accounts for resistance to six different chemical exposures, aggregated via a sixth-order geometric mean:

6 $$\:\begin{array}{c}{C}_{D}=\sqrt[6]{{C}_{1}\cdot\:{C}_{2}\cdot\:{C}_{3}\cdot\:{C}_{4}\cdot\:{C}_{5}\cdot\:{C}_{6}}\:\end{array}$$

Where:

- $$\:{C}_{1}:\:{\text{R}\text{e}\text{s}\text{i}\text{s}\text{t}\text{a}\text{n}\text{c}\text{e}\:\text{t}\text{o}\:\text{w}\text{a}\text{t}\text{e}\text{r}\:(R}_{w})$$

- $$\:{C}_{2}:\text{R}\text{e}\text{s}\text{i}\text{s}\text{t}\text{a}\text{n}\text{c}\text{e}\:\text{t}\text{o}\:\text{a}\text{c}\text{i}\text{d}\text{s}\:\left({R}_{a}\right)$$

- $$\:{C}_{3}:\:\text{R}\text{e}\text{s}\text{i}\text{s}\text{t}\text{a}\text{n}\text{c}\text{e}\:\text{t}\text{o}\:\text{a}\text{l}\text{k}\text{a}\text{l}\text{i}\text{s}\:\left({R}_{k}\right)$$

- $$\:{C}_{4}:\:\text{R}\text{e}\text{s}\text{i}\text{s}\text{t}\text{a}\text{n}\text{c}\text{e}\:\text{t}\text{o}\:\:\text{f}\text{u}\text{e}\text{l},\:\text{o}\text{i}\text{l}\text{s},\:\text{s}\text{o}\text{l}\text{v}\text{e}\text{n}\text{t}\text{s}\:\left({R}_{f}\right)$$

- $$\:{C}_{5}:\:\text{R}\text{e}\text{s}\text{i}\text{s}\text{t}\text{a}\text{n}\text{c}\text{e}\:\text{t}\text{o}\:\text{a}\text{l}\text{c}\text{o}\text{h}\text{o}\text{l}\text{s},\:\text{a}\text{l}\text{d}\text{e}\text{h}\text{y}\text{d}\text{e}\text{s},\:\text{k}\text{e}\text{t}\text{o}\text{n}\text{e}\text{s}\:\left({R}_{h}\right)$$

- $$\:{C}_{6}:\text{R}\text{e}\text{s}\text{i}\text{s}\text{t}\text{a}\text{n}\text{c}\text{e}\:\text{t}\text{o}\:\:\text{U}\text{V}\:\text{R}\text{a}\text{d}\text{i}\text{a}\text{t}\text{i}\text{o}\text{n}\:\left({R}_{u}\right)$$

#### Environmental performance

The environmental performance $\:{(E}_{p})$ is computed as the inverse ratio between the ideal environmental impact $\:{(E}_{x})$ and the impact of the candidate material $\:{(E}_{i}):$

7 $$\:\begin{array}{c}{E}_{p}={E}_{x}/{E}_{i}\end{array}$$

The environmental impact includes the emissions during extraction, processing, and combustion:

8 $$\:\begin{array}{c}{E}_{i}={D}_{1}\:{+\:D}_{2}{\:+\:D}_{3}\:\end{array}$$

Where:

- $$\:{D}_{1}:\:\text{C}\text{O}2\text{e}\:\text{f}\text{o}\text{o}\text{t}\text{p}\text{r}\text{i}\text{n}\text{t}\:\text{p}\text{r}\text{i}\text{m}\text{a}\text{r}\text{y}\:\text{p}\text{r}\text{o}\text{d}\text{u}\text{c}\text{t}\text{i}\text{o}\text{n}\:\left({C}_{p}\right)$$

- $$\:{D}_{2}:\:\text{C}\text{O}2\text{e}\:\text{f}\text{o}\text{o}\text{t}\text{p}\text{r}\text{i}\text{n}\text{t}\:\text{m}\text{a}\text{t}\text{e}\text{r}\text{i}\text{a}\text{l}\:\text{p}\text{r}\text{o}\text{c}\text{e}\text{s}\text{s}\text{i}\text{n}\text{g}\:\left({C}_{m}\right)$$

- $$\:{D}_{3}:\:\text{C}\text{o}\text{m}\text{b}\text{u}\text{s}\text{t}\text{i}\text{o}\text{n}\:\text{C}\text{O}2\text{e}\:\text{f}\text{o}\text{o}\text{t}\text{p}\text{r}\text{i}\text{n}\text{t}\:\left({C}_{c}\right)$$

### Definition of parameters

This section provides a detailed explanation of the key parameters involved in calculating the SDP indicator. Each factor was defined and broken down into measurable variables used in the evaluation process. Table 2 summarizes the calculation parameters corresponding to each durability and environmental factor.

**Table 2:** Definition of calculation parameters for SDP factors.

Factor	Parameter	Calculating parameters	Units	Description
Mechanical Durability ($\:{M}_{D})$	$\:{A}_{1}$	$\:{\sigma\:}_{y}$: Yield strength	$\:MPa$	Maximum stress a material can withstand before it begins to deform plastically.
	$\:{A}_{2}$	$\:{S}_{f}$: Fatigue strength	$\:MPa$	Maximum stress for repeated cycles without cracking or failure.
	$\:{A}_{3}$	$\:E$ : Young modulus	$\:MPa$	A measure of the material’s stiffness, indicating resistance to elastic deformation under stress.
Thermal Durability ($\:{T}_{D})$	$\:{B}_{1}$	$\:{T}_{max}$: Max service temperature	$^\circ{\rm C}$	The highest temperature a material can withstand without structural or functional degradation.
	$\:{B}_{2}$	$\:{T}_{min}$: Min service temperature	$^\circ{\rm C}$	The lowest temperature a material can withstand without becoming brittle or failing.
	$\:{B}_{3}$	Flammability	-	The material’s resistance to ignition and its ability to limit flame propagation.
Chemical Durability ($\:{C}_{D})$	$\:{C}_{1}$	$\:{R}_{w}$: Resistance to water	-	The ability to resist degradation, swelling, or weakening when in contact with water.
	$\:{C}_{2}$	$\:{R}_{a}:\:$Resistance to acids	-	Resistance to corrosion, weakening, or structural changes in acidic environments.
	$\:{C}_{3}$	$\:{R}_{k}$: Resistance to alkalis	-	The capacity to withstand exposure to basic (alkaline) substances without damage.
	$\:{C}_{4}$	$\:{R}_{f}:\:$Resistance to fuel, oils, solvents	-	The ability to maintain integrity and resist degradation when exposed to chemical solvents.
	$\:{C}_{5}$	$\:{R}_{h}\:$: Resistance to alcohols, aldehydes, ketones	-	Resistance to chemical attack or degradation from exposure to organic solvents.
	$\:{C}_{6}$	$\:{R}_{u}:\:$Resistance to UV Radiation	-	The ability to resist damage such as fading, cracking, or weakening caused by ultraviolet exposure.
Environmental Impact ($\:{E}_{p})$	$\:{D}_{1}$	$\:{C}_{p}\::\:$CO2 footprint primary production	$\:kgC{O}_{2}eq/kg$	The amount of CO2 emitted into the environment per kilogram of material extracted.
	$\:{D}_{2}$	$\:{C}_{m}\::\:$CO2 footprint material processing	$\:kgC{O}_{2}eq/kg$	The amount of CO2 emitted into the environment per kilogram of material processed.
	$\:{D}_{3}$	$\:{C}_{c}\::$ Combustion CO2 footprint	$\:kgC{O}_{2}eq/kg$	The amount of CO2 emitted into the environment per kilogram of material taken to combustion.

#### Durability performance ($\:{D}_{p}$)

Durability Performance is a holistic indicator that combines mechanical, thermal, and chemical durability metrics into a single value, aiding material selection at the basic design stage. It is defined as the ratio between the durability performance of a material$\:\:i$ and the ideal X Material. $\:{D}_{p}$ varies from 0.0 to 1.0. Where 1 implies the maximum ideal value for all mechanical, thermal, and chemical durabilities.

##### Mechanical durability ($\:{\varvec{M}}_{\varvec{D}})$

This factor quantifies the resistance of a material to mechanical loads under both static and dynamic conditions, providing insight into its ability to maintain structural integrity over time. A higher level of mechanical durability typically suggests an extended service life because the material can better resist fracture, crack initiation, and undesired plastic deformation.

##### Thermal durability ($\:{\varvec{T}}_{\varvec{D}})$

 This factor evaluates a material’s resistance to thermal stress, including high and low temperatures and rapid fluctuations typical of outdoor environments. Thermal durability ensures materials resist degradation and dimensional changes under extreme heat or cold. Higher values indicate better resistance to thermal degradation, lower thermal expansion, and greater fire resistance. Flammability was rated on a 1 to 4 scale. The full scale is presented in Table A1 – Appendix A.

##### Chemical durability ($\:{\varvec{C}}_{\varvec{D}})$

This factor evaluates the ability of a material to withstand various environmental stressors, including exposure to acidic, alkaline, and alcoholic solutions, as well as UV radiation, water, and solvents. It considers chemical interactions that may degrade the material, making chemical durability key to long-term performance. A standardized 1 to 4 scale was used for all factors. Detailed scales are shown in Table A2 – Appendix A.

The mechanical, thermal, and chemical durability factors in the SDP framework are interrelated, as they collectively influence a material’s overall performance and lifespan in specific applications. For instance, mechanical durability, such as tensile strength and fatigue resistance can be affected by thermal stress, which may cause material degradation or dimensional changes, potentially leading to crack initiation or loss of structural integrity. Likewise, chemical durability, such as resistance to corrosion or UV radiation, helps preserve surface integrity, thereby supporting mechanical performance over time. Thermal durability also influences chemical stability, as elevated temperatures can accelerate reactions like oxidation that degrade both chemical and mechanical properties. These interactions are complex and highly context-dependent, making direct quantification difficult without extensive experimental data for each material and use scenario.

Within the SDP framework, each durability factor is weighted based on application-specific requirements (guided by the 21 evaluation questions) to reflect its relative importance, but the current methodology does not explicitly model their reciprocal effects due to the variability across materials and applications. Instead, the SDP integrates these factors into a single score to facilitate comparative analysis, as demonstrated in the case studies. Future research could explore quantitative models of these interactions using accelerated aging tests to provide more precise predictions of lifespan.

#### Environmental impact ($\:{\varvec{E}}_{\varvec{p}})$

This factor evaluates the material’s overall environmental burden, considering greenhouse gas emissions and other environmental impacts associated with its entire lifecycle. The carbon footprint specifically measures the amount of carbon dioxide equivalent released into the atmosphere throughout the stages of material extraction, processing, manufacturing, and end-of-life recycling or disposal. $\:{E}_{p}$ varies from 0.0 to 1.0. where 1 is the minimum ideal value of the carbon footprint.

We chose carbon footprint as the sole metric in this study due to its significance as a key climate change indicator and the availability of consistent data on emissions for all evaluated alternatives. However, we acknowledge that this approach simplifies environmental impact assessment, as a full LCA would normally encompass multiple impact categories beyond emissions. By focusing on carbon footprint, our metric specifically reflects carbon performance, this provides a clear and meaningful comparison of impacts across options, but it does not capture other important environmental categories.

To ensure transparency and reproducibility in the calculation of carbon footprint values, material can be obtained from established LCA databases and material handbooks. Primary data sources include Ecoinvent 3.8, GaBi, and open-access Environmental Product Declaration (EPD) databases, which provide comprehensive life cycle inventory data for a wide range of materials. Where database values were unavailable, material handbooks, such as those published by the International Energy Agency (IEA) and industry-specific reports, can be consulted. The selection and application of these values should follow standardized methodologies, including ISO 14040/14044 for general LCA principles and EN 15804 for construction materials (for instance), to ensure consistency and comparability. Sector-specific methodological variations, such as those for manufacturing or agricultural products, need to be consider into account for differences in production processes and regional data availability. Assumptions made during data selection, such as the use of global average values for certain materials, must be included during the implementation of this indicator.

Additionally, the importance of mechanical, thermal, and chemical durability for each case study evaluated ($\:{w}_{m}$, $\:{w}_{t},\:$and $\:{w}_{c}$), were obtained by answering a set of 21 yes-or-no questions related to the considered durabilities as shown in the following list. The weight for each type was calculated as the proportion of “yes” responses it received out of the total number of “yes” responses in the questionnaire, ensuring that the weights sum to 1.0. The formula used is:

$$\:{w}_{i}=\frac{{N}_{yes,i}}{{N}_{yes,total}}$$

Where $\:{\text{N}}_{\text{y}\text{e}\text{s},\text{i}}$ is the number of “yes” answers for durability type i, and $\:{\text{N}}_{\text{y}\text{e}\text{s},\text{t}\text{o}\text{t}\text{a}\text{l}}$ is the total count of all “yes” responses across the questionnaire in that case. This approach ensures that the weights reflect the relative importance of each durability factor based on the specific application requirements. The interpretation of the questions is left to the judgment of the designer or development team, considering the functional, environmental, and design priorities of each case. A Python code is provided as supplementary material with the algorithm to easily answer all the 21 questions.

**Q1.** Does the application require the material to bear heavy or static loads without permanent deformation, emphasizing the need of a yield strength suitable for demanding conditions?

**Q2.** Is maintaining structural integrity under stress critical for the material, requiring yield strength adequate for the application’s stress conditions?

**Q3.** Will the material be subjected to repeated or cyclic stress, necessitating fatigue strength sufficient for the application’s cyclic loading conditions?

**Q4.** Is the longevity of the material under cyclic loading conditions a critical factor in its selection?

**Q5.** Does the application demand substantial rigidity, requiring a Young’s modulus that ensures minimal deformation under load?

**Q6.** Is the material required to resist elastic deformation under load, requiring a Young’s modulus appropriate for the application’s stiffness demands?

**Q7.** Is the material required to resist elastic deformation under load, necessitating a Young’s modulus suitable for the application’s stiffness requirements?

**Q8.** Will the material be exposed to temperatures approaching or exceeding its maximum service temperature?

**Q9.** Is it critical to prevent the material from becoming brittle under the application’s minimum temperature conditions?

**Q10.** Will the material be used in environments where temperatures may drop near or below its minimum service temperature?

**Q11.** Will the material be used in environments with a risk of fire, requiring flame resistance suitable for the application’s safety standards?

**Q12.** Will the material undergo regular thermal cycling between its maximum and minimum service temperatures?

**Q13.** Is managing or minimizing thermal expansion and contraction between the max and min service temperatures important?

**Q14.** Does the material need to resist damage from rapid temperature changes between its max and min service temperatures?

**Q15.** Is it crucial for the material to maintain its integrity and properties when exposed to water or humid environments?

**Q16.** Will the material be exposed to acidic environments or substances, necessitating acid resistance?

**Q17.** Is alkali resistance important due to potential exposure to basic or alkaline substances?

**Q18.** Does the application involve contact with fuels, oils, or solvents, requiring the material to be resistant to these substances?

**Q19.** Will the material be exposed to alcohol, aldehydes, or ketones, making resistance to these chemicals necessary?

**Q20.** Is the material expected to be exposed to UV radiation and hence needs to maintain its properties under such exposure?

**Q21.** Does the application require the material to be resistant to multiple chemical agents simultaneously (e.g., water and acids)?

## Case studies

**Fig. 3:**
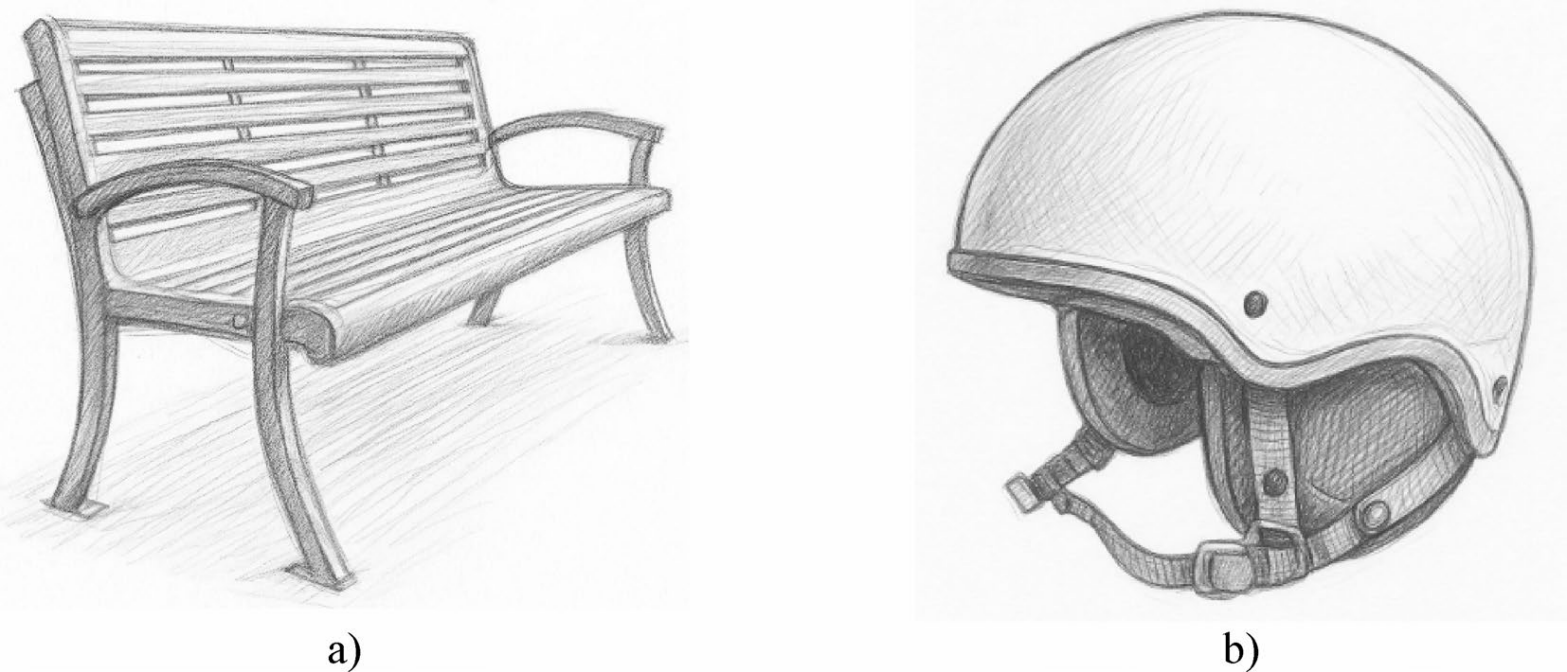
Illustrative images of the case studies presented: (**a**) Outdoor public seating featured in the first case study. (**b**) High-performance sports helmet featured in the second case study. Images drawn by one of the authors (Jenaan Tarabein).

In this section, two case study approaches are introduced to evaluate the accuracy and applicability of the SDP indicator in guiding material selection. Each case presents unique challenges and requirements that enable a comprehensive analysis of the usefulness of the indicator. To facilitate comparison, an “X material” was conceptualized in both cases as an ideal benchmark. The SDP is primarily intended for applications where use-phase impacts are minimal or similar across material options. This scope ensures that material durability and embodied impacts drive the sustainability outcome. In cases where long-term use may involve operational energy or periodic maintenance, those factors should be assessed separately alongside the SDP. While the proposed properties vary based on the context of each case, they serve as a reference point to assess how well the indicator performs under different conditions, which were evaluated for the possible designs of the products shown in Fig. 3. In this study, all parameter values were obtained from the Handbook of Polymer Materials^40^, the World Resource Institute^41^, and Circular Ecology database^42^. These sources were selected for their extensive datasets, detailed information on material properties and manufacturing processes, and robust validation methods. For specific case studies, they provided comprehensive coverage of the parameters required for the SDP assessment. Nonetheless, variations in data quality may occur due to differences in methodologies, regional production processes, or system boundaries, which should be considered when interpreting the results.

The selected materials for both case studies were chosen intentionally to demonstrate two distinct capabilities of the SDP indicator: comparing fundamentally different material families and differentiating closely related materials within the same family, and different approaches for creation of material X. In Case Study I, materials were selected across a broad range: metals (steel, cast iron and aluminum), a polymer (polyethylene (PE)), a conventional composite (concrete), advanced composites (carbon fiber reinforced polymer (CFRP), and Glass fiber reinforced polymer (GFRP)) and a natural material (teak), to showcase the indicator’s versatility in evaluating diverse options. While some choices (such as CFRP) are not common in typical public seating due to cost and complexity, their inclusion serves as boundary cases to test the indicator’s effectiveness across an extensive performance and sustainability spectrum. Conversely, in Case Study II, all chosen materials were from the polymer family to illustrate the indicator’s ability to differentiate subtle but critical performance and sustainability differences among closely related materials, and demonstrate another way to create and analyze material X, facilitating fine-grained material selection within a constrained scope.

### Case study approach I. material X was created from the evaluated materials for outdoor public seating

The development of outdoor public seating exemplifies the equilibrium among durability, functionality, and aesthetic appeal. These installations must endure continuous exposure to environmental factors such as ultraviolet radiation, precipitation, and temperature fluctuations, while fulfilling multiple public functions. In addition to providing a comfortable space for rest and social interaction, the design must satisfy several strict performance criteria. It must provide long-term durability, resist the wear and tear of frequent public use, and withstand constant exposure to outdoor conditions. Furthermore, public seating must offer effective protection against vandalism, with materials sufficiently robust to resist scratching, painting, and burning. In terms of usability, the design must balance aesthetic integration with the surrounding urban environment and functional comfort. Safety standards require that all components be toxin-free. Finally, environmental sustainability remains a key objective, preferring materials with a lower environmental impact throughout their entire life cycle, from production to end of use.

To identify the most suitable material configuration, seven candidate materials were analyzed concurrently, each selected for its potential to meet the demanding requirements of outdoor public seating. GFRP was considered for its lightweight nature, high strength-to-weight ratio, and excellent corrosion resistance. PE offered notable advantages in terms of environmental durability, manufacturing versatility, and adaptability in shaping and coloring. Steel, widely used in public infrastructure, was valued for its superior mechanical strength and long-term structural stability. In parallel, CFRP was assessed for its exceptional tensile strength, low density, and outstanding fatigue resistance. Ductile cast iron was studied for its robustness, ductility, and fatigue performance in load-bearing applications. Concrete was included as a conventional composite material commonly used in public furniture due to its high compressive strength and long service life. Aluminum was analyzed for its favorable balance of strength, corrosion resistance, and light weight, which make it a popular choice in outdoor structures. Finally, teak wood was considered as a natural material, appreciated for its inherent weather resistance, durability, and aesthetic qualities.

 From a comparative analysis of these materials, “Material X” was conceptualized as a reference composite designed to integrate the most advantageous properties of the materials studied. It serves as a benchmark for evaluating performance through the SDP indicator, supporting the development of multifunctional, safe, and sustainable public seating solutions for urban and suburban environments. Table 3 shows the responses to all 21 durability questions with justifications. Table A3 – Appendix A details the parameters used to calculate the SDP, including mechanical, thermal, chemical, and environmental factors for the materials studied. Table A4 – Appendix A presents the resulting durability scores, while Table A5 – Appendix A shows results from the code that determines the significance or weight of each factor under the specific conditions of the case study. Table 4 consolidates these results, showing durability and environmental performance, and the final SDP values for all eight materials (excluding material X).

**Table 3:** Durability evaluation results for case study approach I.

Question Number	Answer (Yes/No)	Justification
Q1	Yes	Must support heavy loads without deforming, ensuring long-term use.
Q2	Yes	Structural integrity is essential under constant stress and weather.
Q3	Yes	Must resist fatigue from repeated stress and environmental exposure.
Q4	Yes	Withstands cyclic loads to avoid frequent replacements.
Q5	Yes	High rigidity ensures user support and comfort.
Q6	No	Some flexibility enhances comfort and prevents brittleness.
Q7	Yes	Safety margin prevents failure under stress and cyclic loading.
Q8	Yes	Must endure outdoor temperatures near service limits.
Q9	Yes	Needs to avoid brittleness and cracking in cold weather.
Q10	Yes	Must perform near or below its minimum service temperature.
Q11	Yes	Fire resistance is vital for public safety.
Q12	Yes	Must withstand thermal cycling without degrading.
Q13	Yes	Controls expansion/contraction to prevent damage.
Q14	Yes	Resists thermal shock to maintain integrity and ensure the material’s integrity.
Q15	Yes	Must retain properties despite moisture exposure.
Q16	Yes	Acid resistance ensures durability in outdoor settings.
Q17	Yes	Must resist alkalis from soil or cleaning agents.
Q18	No	Fuel/oil exposure is minimal and non-critical.
Q19	No	Contact with alcohols or ketones is unlikely.
Q20	Yes	UV resistance is key to prevent degradation.
Q21	Yes	Must resist multiple chemicals in outdoor conditions simultaneously.

**Table 4:** Consolidated calculations of performance and SDP for case study approach I.

Factor	GFRP	CFRP	PE	Steel	Cast Iron	Concrete	Aluminum	Teak	X
$\:D$	147.18	234.87	48.93	364.14	371.27	124.75	248.69	158.73	641.83
$\:{D}_{p}$	0.23	0.37	0.08	0.57	0.58	0.19	0.39	0.25	1.00
$\:{E}_{i}$	8.42	54.91	4.67	4.09	4.06	0.81	17.08	3.74	0.74
$\:{E}_{p}$	0.09	0.01	0.16	0.18	0.18	0.92	0.04	0.2	1.00
$\:SDP$	0.13	0.03	0.1	0.28	0.28	0.32	0.08	0.22	1.00

### Case study approach II. material X was created from the best properties of the polymer family for the exterior of a high-performance sports helmet

The design of the exterior shell of a high-performance sports helmet exemplifies the complex balance between safety, comfort, and durability. Helmets intended for high-impact activities must deliver superior protection while minimizing weight to alleviate user fatigue during prolonged use. These helmets are required to endure extreme conditions, including frequent mechanical stress, exposure to ultraviolet radiation, and fluctuating temperatures, all while complying with strict safety standards and ensuring optimal performance in dynamic environments. It must also meet international safety standards and guarantee durability and reliability to protect users in the long term. Given the increasing concern about environmental sustainability, the development process emphasizes the use of materials that are highly recyclable to minimize the environmental footprint throughout the product’s life cycle.

To address these complex requirements, a range of 5 polymers was considered, including Acrylonitrile Butadiene Styrene (ABS), Polyamide (PA), Polylactide (PLA), Polypropylene (PP), and Polycarbonate (PC). Each material offers distinct attributes: ABS is renowned for its toughness and impact resistance, whereas PA provides strength, wear resistance, and temperature stability. PLA, a biodegradable polymer, was assessed for its eco-friendliness and mechanical properties. PP was evaluated for its lightweight nature and resistance to chemical degradation, and PC, recognized for its high impact resistance was reviewed for its potential to enhance protection.

Unlike the initial case study, where Material X incorporated the best attributes of various materials, the “X material” in this context represents an ideal composite derived from the maximum achievable properties across the polymer family. It serves as a benchmark for assessing the effectiveness of the SDP indicator, guiding the development of a helmet exterior that balances protection, weight, and environmental sustainability.

 The following tables present the results of the second case study of high-performance sports helmets. Table 5 presents the results and justifications for the 21 durability evaluation questions for the specific context and objectives. Table A6 – Appendix A outlines the key parameters used to calculate the SDP, detailing the mechanical, thermal, chemical durability, and environmental impact factors. Table A7 – Appendix A shows the relative importance of the durability factors, displaying the results that identified the contribution of each factor under specific conditions. The calculation tables for this second case study are presented in Table A8 – Appendix A, which provides a summary of the calculation factors for the SDP, and Table 6, which presents the consolidated performance calculations for all five materials (excluding material X) and the final specific durability performance results.

**Table 5:** Durability evaluation results for case study approach II.

Question Number	Answer (Yes/No)	Justification
Q1	Yes	Must resist permanent deformation from high or static loads.
Q2	Yes	Structural integrity ensures protection under stress.
Q3	Yes	Needs fatigue strength for repeated minor impacts and vibration.
Q4	Yes	Durability under cyclic loading ensures long-term safety.
Q5	Yes	Rigid shell disperses force and maintains shape.
Q6	Yes	Maintains structural shape during impact.
Q7	Yes	High safety margin covers unpredictable stresses.
Q8	Yes	Must stay stable at high temperatures.
Q9	Yes	Needs to resist brittleness in cold climates.
Q10	Yes	Must remain stable at sub-zero temperatures.
Q11	No	Fire exposure is not expected.
Q12	Yes	Must endure thermal cycling from outdoor conditions.
Q13	Yes	Needs to resist deformation from expansion.
Q14	Yes	Must handle rapid temperature changes without damage.
Q15	Yes	Water resistance is needed for weather exposure.
Q16	No	Acid exposure is unlikely.
Q17	No	Exposure to alkaline substances is not typical.
Q18	Yes	May contact fuels, oils, or lubricants, especially in transport or handling.
Q19	Yes	Must resist alcohol-based cleaners.
Q20	Yes	Needs UV resistance to prevent degradation.
Q21	Yes	Must resist permanent deformation from high or static loads.

**Table 6:** Consolidated calculations of performance and SDP for case study approach II.

Factor	ABS	PP	PA	PC	PLA	X
$\:D$	41.00	29.45	56.38	67.62	44.30	129.04
$\:{D}_{p}$	0.318	0.228	0.437	0.524	0.343	1.000
$\:{E}_{i}$	6.72	5.67	12.91	9.75	4.32	2.36
$\:{E}_{p}$	0.351	0.416	0.183	0.242	0.547	1.000
$\:SDP$	0.334	0.295	0.258	0.331	0.422	1.000

**Fig. 4:**
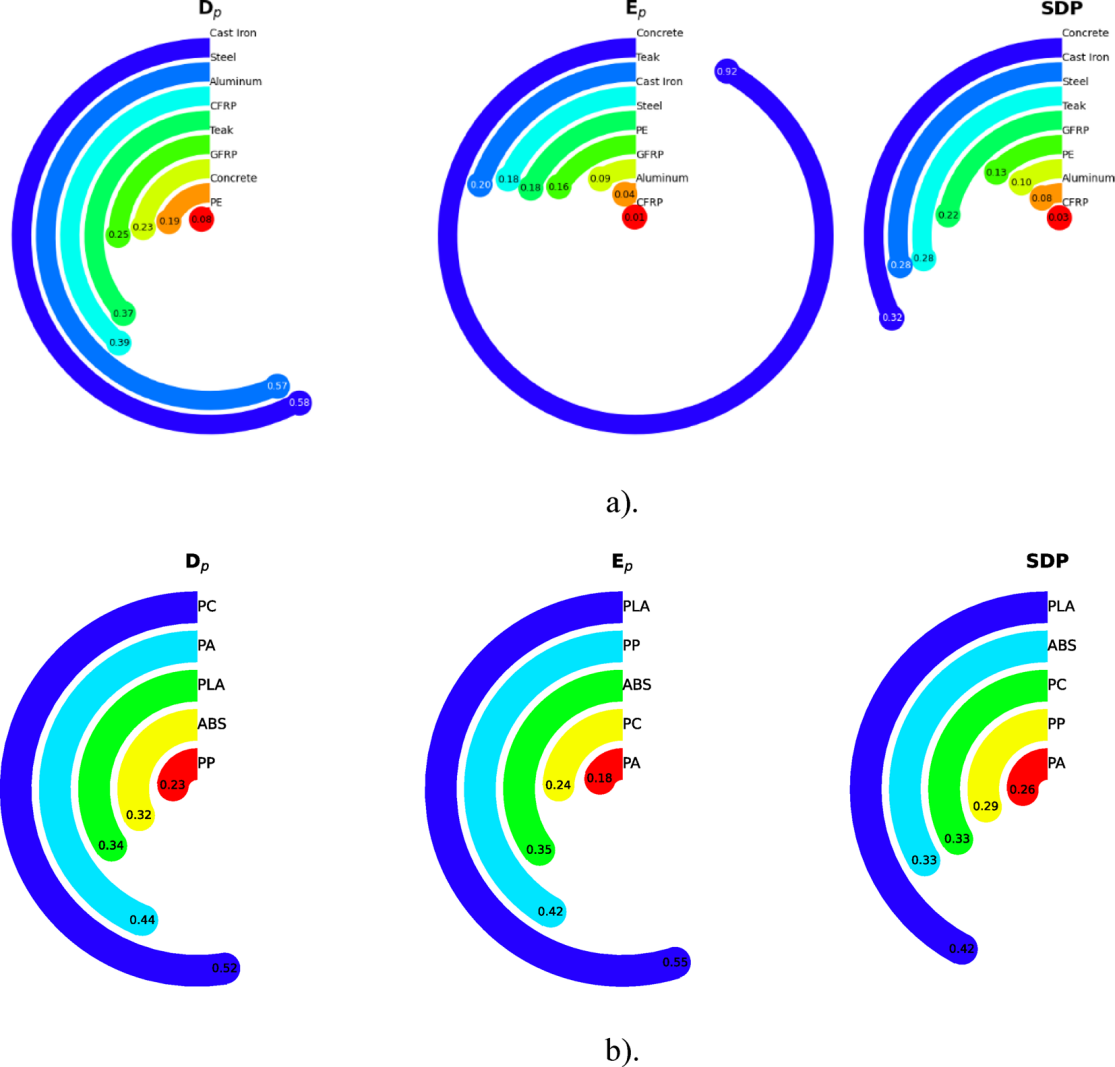
Graphical results of SDP for the case study approaches. **a**) Approach (I) Overall, SDP values indicate that steel offers the highest performance. **b**) Approach (II) Overall SDP values, indicating that PLA offers the highest performance a). b).

Finally, Fig. 4 presents the graphical results of the SDP analysis for the case study under both proposed approaches, complementing the numerical data reported in Tables 4 and 6.

### Expected lifespan under typical use conditions

This section estimates service‑life ranges for each material under the exposure conditions of the two case studies. The SDP score is a dimensionless comparative indicator and is not a measure of years. We therefore combine: (1) SDP inputs that identify durability‑relevant attributes for the case exposure, and (2) published service‑life data that match those exposures and typical protective regimes. The resulting lifespan ranges are literature‑based and are reported together with their assumptions.

In Case Study I, the evaluated materials are subjected to continuous outdoor stressors, including ultraviolet (UV) radiation, thermal cycling, and mechanical wear. The SDP is employed here as a comparative indicator of material durability, but it is not a direct predictor of service life in years. Higher SDP scores reflect more favorable combinations of durability-related attributes, while estimated lifespans are derived from published literature on material performance. The following discussion therefore contextualizes SDP scores with empirical service-life data, assuming typical outdoor exposure.

Concrete demonstrates the longest expected service life, with multiple sources reporting typical values of 50–80 years under normal conditions^43^. Steel also exhibits high durability, with lifespans often extending several decades when corrosion protection is provided; in harsher environments without advanced coatings, values of 20–30 years are more representative^44^. Cast iron shows similar performance, as historical infrastructure records confirm that many components remain in service well beyond their original design lives, supporting lifespans on order of multiple decades^45^. Teak, recognized for its exceptional natural resistance to decay, has been shown to undergo minimal biological degradation over decades, suggesting service lives of 20–30 years or longer in outdoor use^46^.

By contrast, polymer-based materials are more vulnerable to UV and thermal degradation. GFRP typically experiences notable reductions in mechanical properties within the first decade of exposure, with estimated lifespans ranging from 10 to 20 years depending on environmental severity^47^. PE is comparatively more stable but still degrades under prolonged UV radiation; studies of high-density polyethylene (HDPE) liners indicate service lives of approximately 10–20 years in exposed outdoor conditions. Aluminum alloys demonstrate variable performance: clad alloys maintain high durability with multi-decade service lives, while unprotected high-copper alloys may experience severe corrosion within 10–20 years in coastal or industrial atmospheres^48^.

Finally, CFRP illustrates the distinction between SDP and practical service life. Despite receiving the lowest SDP score among the evaluated materials (0.03), largely due to high embodied energy and limited end-of-life recovery options, CFRP exhibits outstanding in-use mechanical stability. Long-term studies report that CFRP laminates can retain over 70% of their tensile strength after 75 years of simulated outdoor exposure^49^. Consequently, CFRP elements may achieve service lives of 50–75 years, highlighting that while the SDP functions as a sustainability-oriented durability index, it should not be interpreted as a direct predictor of chronological service life.

Across these cases, materials with higher SDP scores tend, under comparable exposure and protection assumptions, to align with longer service‑life ranges reported in the literature. Notable exceptions exist when environmental impacts dominate the SDP or when protective regimes strongly influence service life, as illustrated by CFRP. CFRP represents a particular case where low SDP values contrast with long service potential, highlighting that the SDP should be interpreted as a comparative screening tool for material selection, complemented by empirical data when estimating real world lifespans.

In Case Study II, the evaluated materials are subjected to intermittent mechanical impacts, ultraviolet (UV) exposure, and temperature fluctuations. As in Case Study I, the SDP functions as a comparative index, highlighting relative material performance, while lifespan estimates are derived from published service-life and aging studies. The estimates provided here therefore combine SDP rankings with empirical durability data, under the assumption of typical intermittent outdoor use.

PLA, with an SDP score of 0.422, shows favorable environmental performance but limited mechanical and thermal resistance. Recent studies confirm that PLA undergoes significant hydrolytic and thermal degradation within a decade, particularly under fluctuating environmental conditions, supporting service lives of approximately 5–13 years^50^. PC, with an SDP score of 0.331, demonstrates superior impact resistance and transparency, yet artificial weathering studies demonstrate that long term UV exposure leads to embrittlement and yellowing, with expected service lives of 8–12 years under outdoor conditions^51^. PA, with an SDP score of 0.258, benefits from high toughness and abrasion resistance, although moisture absorption can accelerate property loss; experimental studies suggest lifespans on the order of 8–12 years, depending on exposure levels^52^.

PP, with an SDP score of 0.295, has comparatively lower resistance to thermal and photodegradation, leading to lifespans of 5–10 years under outdoor conditions^52^. ABS, with an SDP score of 0.334, provides good impact resistance but shows progressive embrittlement and strength loss under long term UV exposure and thermal cycling; studies of aged ABS components, such as protective helmets, indicate lifespans within the range of 5–10 years^53^.

**Fig. 5:**
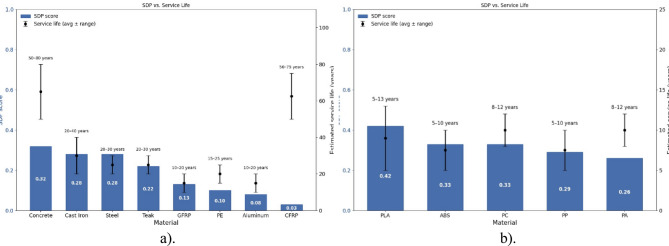
Relationship between SDP scores and estimated service life for the materials analyzed. (**a**) Case Study I, subjected to continuous outdoor stressors. (**b**) Case Study II, exposed to intermittent mechanical and environmental stressors. Lifespan values in this figure correspond to protection regimes for materials where protection is typical.

In addition, Fig. 5 compares SDP scores and literature service life for two case studies—(a) Case Study I and (b) Case Study II. Blue bars show SDP (0–1), while black dots with error bars show average service life and range (years). These are different scales: SDP is a context-specific index derived from the 21 case-study questions, not a measure of years. Therefore, the blue bars are not expected to “exceed” or track the service-life lines; a material can have a long service-life range yet a moderate SDP (or vice versa) depending on the prioritized degradation modes in this application. Use them together: SDP indicates fit to the use context, and the dot/line indicates absolute longevity.

## Findings and discussion

The indicator proposed in this study aids in selecting the most suitable alternative material by assessing both durability and environmental performance. Within the framework of the case studies and methodologies presented, we quantified the individual factors of chemical durability, mechanical durability, thermal durability, and environmental performance (expressed through the carbon footprint) for each material analyzed. These values were compared to the corresponding values for the ideal material defined specifically for each case study. This comparative approach facilitates the establishment of a selection criterion based on the proximity of each material’s performance to the properties of an ideal material.

The first case study involved the selection of optimal material for multifunctional outdoor public seating, and the results underscore the differences observed across all the materials considered. steel and cast Iron exhibited the highest durability values, corresponding to 57.0% and 58.0% of the ideal benchmark, respectively. Aluminum and CFRP also showed notable durability, achieving 39.0% and 37.0% of the ideal. Teak and GFRP demonstrated moderate performance at 25.0% and 23.0%, whereas concrete reached 19.0%. In contrast, PE scored significantly lower, attaining only 8.0% of the ideal.

Regarding environmental performance, concrete achieved the best results among the evaluated materials, reaching 92% of the ideal benchmark, followed by teak (20%), cast iron and Steel (both 18%). PE and GFRP demonstrated lower performance at 16% and 9%, respectively, while aluminum and CFRP showed the poorest results, with only 4% and 1% of the ideal, primarily due to the high environmental impacts associated with their production and processing.

By integrating both durability and environmental metrics, concrete emerged as the top performing material among the real alternatives, achieving 32% of the ideal benchmark, closely followed by cast iron and steel at 28% each. Teak also demonstrated relatively balanced performance, reaching 22%. GFRP, PE, and aluminum obtained moderate SDP scores (13%, 10%, and 8%, respectively), whereas CFRP, despite its comparatively high durability, recorded the lowest score at only 3%, highlighting its poor environmental compatibility. Overall, concrete, steel, and cast iron provided the most balanced combination of durability and environmental performance, positioning them as the most suitable candidates relative to the benchmark. Although other materials such as teak, GFRP, and PE may still be considered depending on specific design priorities or constraints, their lower overall performance renders them less favorable.

The inclusion of metals (cast iron, steel, and aluminum), teak and concrete make the case study more representative of real-world practices, as these materials are among the most prevalent in urban furniture manufacturing. The revised SDP results highlight how concrete, along with cast iron, steel, and teak, achieves relatively higher scores and corresponds to materials already widely adopted in outdoor seating applications. This alignment between SDP outcomes and established usage patterns reinforces the validity of the framework, showing that the indicator not only differentiates across diverse material classes but also converges with the materials proven most effective in practice.

The second case study focused on selecting the optimal material for the exterior of a lightweight, high-performance sports helmet. In terms of durability, PC and PA performed the best, achieving 52.4% and 43.7% of the ideal performance, respectively. PLA, ABS, and PP followed with 34.3%, 31.8%, and 22.8% of the ideal. Although PLA was not the most durable, it maintained a competitive position among the top half of the materials in this aspect. Regarding environmental performance, PLA retained the lowest environmental impact, with a normalized score of 0.547. PP followed with 0.416, and ABS with 0.351. PA and PC had significantly lower environmental scores, reflecting their relatively higher environmental burden.

When both factors were integrated into the SDP score, PLA remained the most balanced material, achieving 42.2% of the ideal performance. This confirms its superior overall trade-off between durability and sustainability. PC, despite its high durability, reached an SDP of 33.1%, making it a close second. ABS and PP also showed moderate balances, while PA, though the most durable material, had the lowest SDP due to its poor environmental performance. In conclusion, PLA was the most suitable material for the sports helmet exterior, thanks to its optimal combination of acceptable durability and outstanding environmental performance. While PC, ABS, and PP offer competitive alternatives under certain project-specific conditions, PLA provides the best overall balance, according to the standardized performance index.

The findings of this study have significant implications for material selection in various industries. In the first case study, Material X was defined as the best performing option among all the studied materials considering both durability and environmental impact. This approach enables systematic evaluation and comparison of materials from different families, making the methodology adaptable to a broad range of applications. The use of an ideal material for each family material provides a clear benchmark, helping to select the most suitable material that balances performance and sustainability. In the second case study, Material X served as the ideal benchmark within the polymer family. This approach allows for in-depth evaluation of polymers or any family of materials, making the methodology particularly useful for industries that rely on only one type.

Specifically, for approach one, the introduction of any new superior material into the candidate list would alter the Material X benchmark, consequently rescaling all SDP scores. This sensitivity highlights the importance of careful initial candidate selection and explicit recognition of benchmark dependency when interpreting results.

We acknowledge that our sustainability-durability index, like any composite metric, involves certain shortcomings. First, the index necessarily simplifies complexity, as it cannot capture every detail of sustainability and durability. Second, the accuracy of the index is constrained by the quality and availability of input data. For instance, reliable data on material lifespan, environmental impacts, or reuse/recycling rates can be scarce or uncertain. Third, the scope of the index is presently focused on environmental sustainability and technical durability. It does not include other important factors like economic cost or social impact, since real-world material selection also depends on budget constraints and social factors. In summary, while the proposed index is a helpful step toward integrating durability into sustainable material selection, it has clear limitations regarding data uncertainty, inclusion of other factors, and some generalization.

It is worth discussing the applicability and limits of the SDP in the context of the full product lifecycle. The current SDP model deliberately focuses on material-intrinsic durability and embodied environmental impact and excludes use-phase impacts such as operational energy consumption or maintenance emissions. This focus aligns with the scope of our case studies, where the use phase is not energy-intensive and maintenance requirements are relatively low. We acknowledge that in many long-lifespan product scenarios, use-phase factors like maintenance, repair, or energy use can be significant. For example, extending a steel component’s life might require periodic repainting, which incurs in the use phase. These impacts are not captured in the SDP score, posing a limitation when applying the indicator to cases where substantial maintenance is needed for longevity. In such cases, the SDP should be complemented by a broader analysis: users might use SDP to choose an inherently durable, low-footprint material, and then separately evaluate the environmental trade-offs of the required maintenance or operational impacts.

We have made this scope explicit in the methodology to avoid confusion. Importantly, this does not diminish the value of SDP rather, it clarifies that SDP is intended as an early-stage screening tool for material selection under the assumption of normal use conditions. Future work can integrate additional metrics for maintenance intensity or in-use environmental impacts to expand the SDP’s applicability. In fact, as noted, incorporating factors like maintenance/repair frequency and actual field failure rates could provide a fuller picture of durability in use. For now, users should interpret a high SDP score as indicating a material that is fundamentally well suited for long life provided it is cared for appropriately, and plan for any necessary use-phase support as part of the product’s lifecycle strategy.

We think it is possible to improve the index in future research so that it becomes more accurate, reliable, and useful for its intended users. To enhance accuracy, incorporate additional relevant indicators and more comprehensive data. For example, integrating metrics for maintenance/repairability and actual field failure rates could give a fuller picture of durability in use, and including end-of-life recovery factors would address sustainability more holistically. Future research can extend this index framework by including socio-economic dimensions or linking it with circular economy models, thereby evolving it into a more comprehensive sustainability assessment tool, making the proposed method more robust and valuable for both practitioners and researchers in sustainable design.

## Conclusions

This study presents the SDP indicator, a novel framework for material selection that integrates mechanical, thermal, and chemical durability with environmental impact into a single quantitative score. Unlike existing material selection methods that focus on isolated properties or emphasize recyclability over longevity, the SDP provides a comprehensive evaluation tool that enables engineers and designers to make informed, sustainability-driven choices. The SDP approach aligns with contemporary sustainable design frameworks. For example, it supports the European Commission’s “Safe and Sustainable by Design” (SSbD) initiative, SSbD aims to “minimize the impact on health, climate and the environment during sourcing, production, use and end-of-life”^54^ SDP enables the integration of safety (durability) and sustainability (low environmental impact) considerations from the earliest stages of product design, providing designers with a practical tool to implement SSbD principles from the concept stage. It also complements LCA principles, incorporating carbon footprint as a decision metric, and reinforces circular economy strategies through a focus on material longevity. Employing SDP in early design stages allows engineers to select materials aligned with durability-driven design and regulatory sustainability goals.

By Benchmarking candidate materials against ideal reference material, the SDP offers a structured methodology for selecting materials that maximize durability while minimizing environmental burden. The two cases of study demonstrate the versatility and applicability of the SDP across different product categories. The results confirmed that concrete showed the most balanced performance for the outdoor seating application, achieving approximately 32% of the ideal material’s score, making it the most balanced choice in terms of longevity and environmental impact. In contrast, PLA emerged as the best candidate (42% of the ideal material’s performance) for the exterior of a sport helmet due to its lower carbon footprint and despite PC’s higher durability, illustrating the SDP’s ability to balance durability performance with sustainability metrics.

The findings of this research reinforce the importance of integrating durability and environmental performance when selecting materials for extended lifespan products. In the outdoor seating case, concrete emerged as the most balanced option, outperforming other commonly used materials in this application, such as cast iron, steel, and teak. This result reinforces the relevance of the SDP framework by showing that, among conventional choices, concrete stands out for its ability to combine adequate mechanical, thermal, and chemical resistance with a comparatively lower environmental impact when normalized against the ideal benchmark. Conversely, in the sports helmet case, where weight and sustainability played a larger role, PLA emerged as the most balanced option despite not being the most durable material in absolute terms. These results highlight how context-specific durability weighting enables the SDP to prioritize different material attributes based on the intended application.

While the SDP presents a robust material selection methodology, some limitations must be addressed in future research. The current study relies on carbon footprint as the sole environmental performance metric, which, while essential, does not capture other sustainability considerations such as water use, energy consumption, recyclability, and toxicity. Expanding the SDP framework to incorporate LCA principles and SSbD comprehensive criteria would provide a more global evaluation of material’s long-term impact. Additionally, economic feasibility was not factored into the SDP score. Material cost, processing complexity, and supply chain constraints can significantly influence the viability of sustainable material choices, and their integration into the SDP model would improve its practical applicability.

The complexity of product systems and their life cycle stages continue to challenge accurate carbon footprint assessments. Although our framework offers a flexible approach to data selection, limitations remain, particularly regarding data availability for environmental metrics and material durability properties. The lack of standardized, comprehensive databases and methodological variations (e.g., system boundaries or allocation methods) may affect result consistency. These factors must be considered when interpreting the findings. Future work should focus on enhancing data precision through real-time sources and advanced modeling techniques, aiming to reduce variability and strengthen the framework’s applicability across sectors.

Another key limitation is its focus on production and end-of-life stages, which excludes use-phase environmental impacts, particularly relevant for energy-consuming products. This limits the applicability of the SDP in contexts where operational energy use constitutes a substantial portion of a product’s overall environmental footprint. To address this, future research should prioritize the integration of LCA data specific to operational energy consumption, which extends the SDP’s relevance to energy-intensive applications and enhancing its utility across a wider range of engineering and design scenarios.

It is important to note that in this study $\:{E}_{p}$ reflects only the carbon footprint equivalent (a key climate metric) of each alternative, rather than a full spectrum of environmental impacts. While this focused approach gives a picture of the performance, it remains a simplified representation and has a lot of potential for improvement.

## Supplementary Information

Below is the link to the electronic supplementary material.

Supplementary Material 1

Supplementary Material 2

## Data Availability

All data generated or analyzed during this study are included in this published article, and its supplementary information files.

## Declarations

### Use of IA declaration

During the preparation of this work the author(s) used ChatGPT in order to check grammar and readability. After using this tool/service, the authors reviewed and edited the content as needed and take full responsibility for the content of the publication.
