# Supplementary material for: Proposing a material selection indicator for the design of extended lifespan products
Source: Sci Rep. 2025 Oct 24;15:37331. doi: 10.1038/s41598-025-21186-0 (PMC12552478; doi:10.1038/s41598-025-21186-0)
Supplement: Supplementary file 1 — Supplementary Material 1 [file 41598_2025_21186_MOESM1_ESM.docx]

**Appendix A**

**Table A1.** Quantitative scale for flammability.

| Numeric Value | Flammability |
| --- | --- |
| 1 | Highly Flammable |
| 2 | Slow-Burning |
| 3 | Self-extinguishing |
| 4 | Non-Flammable |

**Table A2.** Quantitative scale for chemical durability.

| Numeric Value | Resistance to Water | Resistance to Acids | Resistance to Alkalis | Resistance to Fuels | Resistance to Alcohols | Resistance to UV |
| --- | --- | --- | --- | --- | --- | --- |
| 1 | Unacceptable | Unacceptable | Unacceptable | Unacceptable | Unacceptable | Poor |
| 2 | Limited Use | Limited Use | Limited Use | Limited Use | Limited Use | Fair |
| 3 | Acceptable | Acceptable | Acceptable | Acceptable | Acceptable | Good |
| 4 | Excellent | Excellent | Excellent | Excellent | Excellent | Excellent |

**Table A3**. Summary of calculation parameters for case study approach I.

| Factor | Parameters | GFRP | CFRP | PE | Steel | Cast Iron | Concrete | Aluminum | Teak | X |
| --- | --- | --- | --- | --- | --- | --- | --- | --- | --- | --- |
| Mechanical Durability ($M_{D})$ | $\sigma_{y}$ (MPa) | 255.5 | 800 | 23.45 | 652.5 | 438 | 2 | 127.5 | 56.7 | 800 |
|  | $S_{f}$ (MPa) | 66.2 | 225 | 22.0 | 389.0 | 256 | 1.15 | 98.6 | 30.25 | 389.0 |
|  | $E$ (Mpa) | 21400 | 114000 | 758.5 | 210000 | 175000 | 35500 | 68300 | 11750 | 210000 |
| Thermal Durability ($T_{D})$ | $T_{max}$(°C) | 220 | 220 | 110 | 350 | 450 | 977 | 150 | 150 | 977 |
|  | $T_{min}$(°C) | -123 | -123 | -123 | -53.2 | -69.2 | -160 | -273 | -273 | -273 |
|  | Flammability | 2 | 2 | 1 | 4 | 4 | 4 | 4 | 4 | 4 |
| Chemical Durability ($C_{D})$ | $R_{w}$ | 4 | 4 | 4 | 3 | 3 | 4 | 4 | 4 | 4 |
|  | $R_{a}$ | 2 | 2 | 4 | 1 | 2 | 2 | 4 | 4 | 4 |
|  | $R_{k}$ | 3 | 3 | 4 | 3 | 3 | 3 | 2 | 1 | 4 |
|  | $R_{f}$ | 3 | 3 | 3 | 4 | 4 | 3 | 4 | 4 | 4 |
|  | $R_{h}:$ | 2 | 2 | 4 | 3 | 3 | 2 | 3 | 3 | 4 |
|  | $R_{u}$ | 2 | 3 | 2 | 4 | 4 | 4 | 4 | 4 | 4 |
| Environmental Impact ($E_{i})$ | $C_{p}$ ($kgCO_{2}/kg$) | 6.32 | 50.5 | 1.95 | 2.49 | 2.49 | 0.128 | 13.9 | 0.578 | 0.128 |
|  | $C_{m}$($kgCO_{2}/kg$) | 1.08 | 1.08 | 1.73 | 0.90 | 0.90 | 0.24 | 2.81 | 1.78 | 0.24 |
|  | $C_{c}$($kgCO_{2}/kg$) | 1.02 | 3.33 | 0.99 | 0.70 | 0.70 | 0.44 | 0.375 | 1.38 | 0.375 |

**Table A4.** Summary of durabilities calculation for case study approach I

| Factor | GFRP | CFRP | PE | Steel | Cast Iron | Concrete | Aluminum | Teak | X |
| --- | --- | --- | --- | --- | --- | --- | --- | --- | --- |
| $M_{D}$ | 712.67 | 2737.74 | 73.14 | 3763.42 | 2697.23 | 43.38 | 950.46 | 272.13 | 4027.97 |
| $T_{D}$ | 686.00 | 686.00 | 233.00 | 1612.80 | 2076.80 | 4548.00 | 1692.00 | 1692.00 | 5000.00 |
| $C_{D}$ | 2.57 | 2.75 | 3.40 | 2.75 | 3.09 | 2.88 | 3.40 | 3.03 | 4.00 |

**Table A5.** Importance of factors for durability performance for case study approach I.

| Factor | Options | Percentage | Importance |
| --- | --- | --- | --- |
| Mechanical Importance ($w_{m})$ | Yes | 85.71% | 0.3333 = 33.33% |
|  | No | 14.29% |  |
| Thermal Importance ($w_{t})$ | Yes | 100% | 0.3889 = 38.89% |
|  | No | 0% |  |
| Chemical Importance ($w_{c})$ | Yes | 71.43% | 0.2778 = 27.78% |
|  | No | 28.57% |  |

**Table A6.** Summary of calculation parameters for case study approach II.

| Factor | Parameters | ABS | PC | PA | PP | PLA | X |
| --- | --- | --- | --- | --- | --- | --- | --- |
| Mechanical Durability ($M_{D})$ | $\sigma_{y}$ (Mpa) | 42.05 | 62.1 | 46.5 | 26.25 | 52.5 | 75 |
|  | $S_{f}$ (MPa) | 17.95 | 27.25 | 21 | 7.83 | 24.95 | 35 |
|  | $E$ (Mpa) | 2415 | 2380 | 1490 | 922 | 3450 | 3500 |
| Thermal Durability ($T_{D})$ | $T_{max}$(°C) | 76.9 | 116 | 130 | 83.9 | 54.9 | 260 |
|  | $T_{min}$(°C) | -45.2 | -47.2 | -80 | -25.2 | -20.2 | -200 |
|  | Flammability | 1 | 2 | 2 | 1 | 2 | 3 |
| Chemical Durability ($C_{D})$ | $R_{w}$ | 4 | 4 | 3 | 4 | 3 | 4 |
|  | $R_{a}$ | 3 | 4 | 1 | 3 | 1 | 4 |
|  | $R_{k}$ | 4 | 3 | 1 | 4 | 1 | 4 |
|  | $R_{f}$ | 2 | 3 | 4 | 2 | 2 | 4 |
|  | $R_{h}:$ | 2 | 3 | 2 | 2 | 1 | 4 |
|  | $R_{u}$ | 1 | 2 | 2 | 1 | 3 | 3 |
| Environmental Impact ($E_{i})$ | $C_{p}$ ($kgCO_{2}/kg$) | 3.77 | 6.18 | 8 | 3.01 | 2.4 | 0.94 |
|  | $C_{m}$($kgCO_{2}/kg$) | 1.32 | 1.46 | 3.19 | 1.66 | 0.82 | 0.32 |
|  | $C_{c}$($kgCO_{2}/kg$) | 1.63 | 2.11 | 1.72 | 1 | 1.1 | 1.1 |

**Table A7.** Summary of durabilities calculation for case study approach II

| Factor | ABS | PC | PA | PP | PLA | X |
| --- | --- | --- | --- | --- | --- | --- |
| $M_{D}$ | 122.16 | 159.10 | 113.31 | 57.44 | 165.33 | 209.44 |
| $T_{D}$ | 122.10 | 326.4 | 420.00 | 109.10 | 150.20 | 1380.00 |
| $C_{D}$ | 2.40 | 3.09 | 1.91 | 2.40 | 1.62 | 3.81 |

**Table A8.** Importance of factors for durability performance for case study approach II.

| Factor | Options | Percentage | Importance |
| --- | --- | --- | --- |
| Mechanical Importance ($w_{m})$ | Yes | 100.00% | 0.3889 = 38.89% |
|  | No | 0.00% |  |
| Thermal Importance ($w_{t})$ | Yes | 85.71% | 0.3333 = 33.33% |
|  | No | 14.29% |  |
| Chemical Importance ($w_{c})$ | Yes | 71.43% | 0.2778 = 27.78% |
|  | No | 28.57% |  |

**Appendix B**

**Sensitivity analysis SDP equation**

The heatmap illustrated in Fig. B1 presents a two-dimensional sensitivity analysis of the SDP value as a function of two independent variables: $D_{p}$ (horizontal axis) and $E_{p}$ (vertical axis). Both parameters range from 0 to 1, with the SDP value represented through a color gradient, where lighter colors indicate higher values. The color bar on the right provides the scale of the SDP value.

The SDP value demonstrates a non-linear yet monotonic increase concerning both variables. The lowest SDP values are observed when both parameters are near zero, while the highest values occur as both parameters approach unity. This suggests that the SDP function is synergistic with respect to both variables, indicating that high values in both are necessary to maximize the output. From a modeling perspective, the function appears to favor configurations where both input parameters are simultaneously high, which may be relevant in applications such as material property optimization, system reliability assessment, or multi-criteria decision-making scenarios.

**
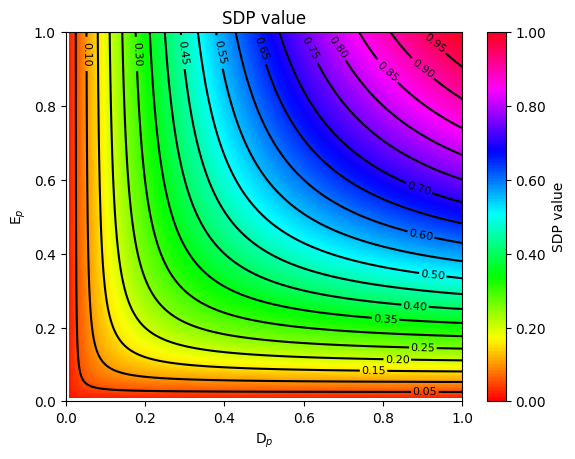
**

**Fig. B1.** Contour plot of the SDP objective function as a function of its parameters, illustrating the sensitivity to potential parameter values.
