# Supplementary material for: Proposing a material selection indicator for the design of extended lifespan products
Source: Sci Rep. 2025 Oct 24;15:37331. doi: 10.1038/s41598-025-21186-0 (PMC12552478; doi:10.1038/s41598-025-21186-0)
Supplement: Supplementary file 2 — Supplementary Material 2 [file 41598_2025_21186_MOESM2_ESM.docx]

import pandas as pd

import numpy as np

import matplotlib.pyplot as plt

import matplotlib.gridspec as gridspec

from matplotlib.patches import Patch

*def* determine_weights():

    print("\n=== Weight Determination Questions ===")

    print("Answer with '1' for yes or '2' for no\n")

    mechanical_questions = [

        "Yield Strength - Load Bearing: Does the application require the material to bear heavy or static loads without permanent deformation, emphasizing the need of a yield strength suitable for demanding conditions?",

        "Yield Strength - Structural Integrity: Is maintaining structural integrity under stress critical for the material, requiring yield strength adequate for the application’s stress conditions?",

        "Fatigue Strength - Will the material be subjected to repeated or cyclic stress, necessitating fatigue strength sufficient for the application’s cyclic loading conditions?",

        "Fatigue Strength - Longevity: Is the longevity of the material under cyclic loading conditions a critical factor in its selection?",

        "Young's Modulus - Rigidity: Does the application demand substantial rigidity, requiring a Young’s modulus that ensures minimal deformation under load?",

        "Young's Modulus - Is the material required to resist elastic deformation under load, requiring a Young’s modulus appropriate for the application’s stiffness demands?",

        "Yield Strength & Fatigue Strength - Safety Margin: DIs the material required to resist elastic deformation under load, necessitating a Young’s modulus suitable for the application’s stiffness requirements?"

    ]

    thermal_questions = [

        "Max Service Temperature - High-Temperature Exposure: Will the material be exposed to temperatures approaching or exceeding its maximum service temperature?",

        "Min Service Temperature - Brittle Behavior: Is it critical to prevent the material from becoming brittle under the application’s minimum temperature conditions?",

        "Min Service Temperature - Low-Temperature Exposure: Will the material be used in environments where temperatures may drop near or below its minimum service temperature?",

        "Flammability - Fire Risk Environments: Will the material be used in environments with a risk of fire, requiring flame resistance suitable for the application’s safety standards?  ",

        "Max & Min Service Temperature - Thermal Cycling: Will the material undergo regular thermal cycling between its maximum and minimum service temperatures?",

        "Thermal Expansion: Is managing or minimizing thermal expansion and contraction between the max and min service temperatures important?",

        "Thermal Shock Resistance: Does the material need to resist damage from rapid temperature changes between its max and min service temperatures?"

    ]

    chemical_questions = [

        "Resistance to Water: Is it crucial for the material to maintain its integrity and properties when exposed to water or humid environments?",

        "Resistance to Acids: Will the material be exposed to acidic environments or substances, necessitating acid resistance?",

        "Resistance to Alkalis: Is alkali resistance important due to potential exposure to basic or alkaline substances?",

        "Resistance to Fuels, Oils, Solvents: Does the application involve contact with fuels, oils, or solvents, requiring the material to be resistant to these substances?",

        "Resistance to Alcohols, Aldehydes, Ketones: Will the material be exposed to alcohols, aldehydes, or ketones, making resistance to these chemicals necessary?",

        "Resistance to UV Radiation: Is the material expected to be exposed to UV radiation, and hence needs to maintain its properties under such exposure?",

        "Combined Chemical Resistance: Does the application require the material to be resistant to multiple chemical agents simultaneously (e.g., water and acids)?"

    ]

*def* ask_questions(*section_name*, *questions*):

        print(*f*"\n{section_name} Section:")

        answers = []

        for question in questions:

            response = input(*f*"{question}\nAnswer with '1' for yes or '2' for no: ").strip()

            while response not in ['1', '2']:

                print("Invalid input. Please answer with '1' for yes or '2' for no.")

                response = input(*f*"{question}\nAnswer with '1' for yes or '2' for no: ").strip()

            answers.append(response)

        return answers

    mechanical_answers = ask_questions("Mechanical", mechanical_questions)

    thermal_answers = ask_questions("Thermal", thermal_questions)

    chemical_answers = ask_questions("Chemical", chemical_questions)

*def* calculate_percentage(*answers*):

        yes_count = answers.count('1')

        return (yes_count / len(answers)) * 100 if len(answers) > 0 else 0

    mechanical_percent = calculate_percentage(mechanical_answers)

    thermal_percent = calculate_percentage(thermal_answers)

    chemical_percent = calculate_percentage(chemical_answers)

    total = mechanical_percent + thermal_percent + chemical_percent

    if total == 0:

        total = 1  # Prevent division by zero

    w_m = mechanical_percent / total

    w_t = thermal_percent / total

    w_c = chemical_percent / total

    print("\nCalculated Weights:")

    print(*f*"Mechanical (w_m): {w_m:.4f}")

    print(*f*"Thermal (w_t): {w_t:.4f}")

    print(*f*"Chemical (w_c): {w_c:.4f}")

    return w_m, w_t, w_c

*def* calcular_parametros(*materiales*, *caso_num*, *w_m*, *w_t*, *w_c*, *calcular_x*=True):

    if calcular_x:

        df_all = pd.DataFrame(materiales)

        min_keys = ["CO2 Combustion", "CO2 Processing",

                   "Min service temperature (°C)", "Footprint - Primary Production"]

        material_x = {}

        for key in df_all.index:

            material_x[key] = df_all.loc[key].min() if key in min_keys else df_all.loc[key].max()

        materiales["Material X"] = material_x

    data_for_calc = {'Material': []}

    for material, props in materiales.items():

        data_for_calc['Material'].append(material)

    df = pd.DataFrame(data_for_calc)

    properties_map = {

        'A1': "Yield strength (MPa)",

        'A2': "Fatigue strength (MPa)",

        'A3': "Young modulus (MPa)",

        'B1': "Max service temperature (°C)",

        'B2': "Min service temperature (°C)",

        'B3': "Flammability",

        'C1': "Resistance to water",

        'C2': "Resistance to acids",

        'C3': "Resistance to alkalis",

        'C4': "Resistance to fuel, oils, solvents",

        'C5': "Resistance to alcohols, aldehydes, ketones",

        'C6': "Resistance to UV Radiation",

        'D1': "Footprint - Primary Production",

        'D2': "CO2 Combustion",

        'D3': "CO2 Processing",

    }

    for code, prop in properties_map.items():

        df[code] = [materiales[material].get(prop, np.nan) for material in df['Material']]

    df['Md'] = (df['A1'] * df['A2'] * df['A3'])**(1/3)

    df['Td'] = (df['B1'] - df['B2']) * df['B3']

    chem_cols = ['C1', 'C2', 'C3', 'C4', 'C5', 'C6']

    df['Cd'] = df[chem_cols].prod(axis=1)**(1/6)

    df['Di'] = (df['Md']**w_m) * (df['Td']**w_t) * (df['Cd']**w_c)

    D_x = df[df['Material'] == 'Material X']['Di'].values[0]

    df['Dp'] = df['Di'] / D_x

    df['Ei'] = df['D1'] + df['D2'] + df['D3']

    E_x = df[df['Material'] == 'Material X']['Ei'].values[0]

    df['Ep'] = E_x / df['Ei']

    df['SDP'] = 2 * df['Dp'] * df['Ep'] / (df['Dp'] + df['Ep'])

    final_columns = ['Material', 'Md', 'Td', 'Cd', 'Di', 'Dp', 'Ei', 'Ep', 'SDP']

    return df[final_columns].sort_values(by='SDP', ascending=False).reset_index(drop=True), materiales

*def* mostrar_propiedades(*materiales*, *caso_num*):

    fig = plt.figure(figsize=(18, 16))

    gs = gridspec.GridSpec(41, 2, figure=fig, wspace=0.5, hspace=1.2)

    for i, (name, props) in enumerate(sorted(materiales.items())):

        row = i // 2

        col = i % 2

        if row >= 4:

            continue

        df_props = pd.DataFrame(props, index=[name]).T

        ax = fig.add_subplot(gs[row, col])

        ax.axis("off")

        table = ax.table(cellText=[[*f*"{name}", ""]] + [[k, v] for k, v in df_props.itertuples()],

                         colWidths=[0.6, 0.4],

                         cellLoc='center',

                         loc='center')

        table.auto_set_font_size(False)

        table.set_fontsize(9)

        table.scale(1.2, 1.2)

        table[(0, 0)].set_text_props(weight='bold', fontsize=10)

    plt.suptitle(*f*"Material Properties - Case {caso_num}", fontsize=20, weight='bold')

    plt.show()

*def* mostrar_resultados(*df_final*, *caso_num*):

    # Crear figura estilizada

    plt.figure(figsize=(12, 8))

    ax = plt.subplot(111, frame_on=False)

    ax.xaxis.set_visible(False)

    ax.yaxis.set_visible(False)

    # Preparar datos y formatear números

    df_display = df_final.copy()

    numeric_cols = ['Md', 'Td', 'Cd', 'Di', 'Dp', 'Ei', 'Ep', 'SDP']

    df_display[numeric_cols] = df_display[numeric_cols].round(2)

    # Crear tabla

    tabla = plt.table(

        cellText=df_display.values,

        colLabels=df_display.columns,

        colColours=['#f3f3f3']*len(df_display.columns),

        cellLoc='center',

        loc='center'

    )

    # Estilizar tabla

    tabla.auto_set_font_size(False)

    tabla.set_fontsize(11)

    tabla.scale(1.2, 1.5)

    # Resaltar encabezados

    for cell in tabla.get_celld():

        if cell[0] == 0:

            tabla.get_celld()[cell].set_text_props(weight='bold', color='white')

            tabla.get_celld()[cell].set_facecolor('#2d4059')

    # Añadir título

    plt.suptitle(*f*'Case {caso_num} - Final Results',

                fontsize=14,

                weight='bold',

                y=0.93,

                color='#2d4059')

    plt.tight_layout()

    plt.show()

*def* generar_grafica_polar(*df_final*, *case_num*):

    df_filtered = df_final[df_final['Material'] != 'Material X'].copy()

    max_val = 1.0

    bar_height = 0.8

    indicators = ['Dp', 'Ep', 'SDP']

    indicator_titles = ['D$_{p}$', 'E$_{p}$', 'SDP']

    if case_num == 1:

        markersize = 24

        radius_offset = 0.01

    else:

        markersize = 29.5

        radius_offset = -0.013

    materials = df_filtered['Material'].unique()

    colors = plt.cm.hsv(np.linspace(0, 0.70, len(materials)))

    fig, axes = plt.subplots(1, 3, figsize=(17, 8), subplot_kw={'projection': 'polar'})

    plt.subplots_adjust(wspace=0.4, bottom=0)

    for idx, (indicador, title) in enumerate(zip(indicators, indicator_titles)):

        ax = axes[idx]

        ax.set_facecolor('white')

        plt.sca(ax)

        plt.axis('off')

        df_sorted = df_filtered.sort_values(by=indicador)

        for i, row in enumerate(df_sorted.iterrows()):

            material = row[1]['Material']

            value = row[1][indicador]

            angle = value * 2 * np.pi / max_val

            radius = i

            color = colors[i]

            ax.barh(radius, angle, height=bar_height, color=color, edgecolor='white', linewidth=0)

            ax.plot(angle, radius + radius_offset, 'o', color=color, markersize=markersize,

                    markeredgecolor='white', markeredgewidth=0)

            offset=0.6

            ax.text(0, radius+offset, material, va='center', ha='left', fontsize=10, color='black')

                # Cambiar color solo en los últimos dos materiales

            if i >= len(df_sorted) - 2:

             num_color = "white"

            else:

             num_color = "black"

            ax.text(angle, radius, *f*"{value:.2f}",

            va='center', ha='center', fontsize=9, color=num_color,fontweight="bold")

        ax.set_yticks(range(len(df_sorted)))

        ax.set_yticklabels(df_sorted['Material'], fontsize=14)

        ax.set_theta_zero_location('N')

        ax.set_theta_direction(1)

        ax.set_xticks([])

        ax.set_title(title, fontsize=18, fontweight='bold', y=1.02)

    plt.suptitle(*f*"Case {case_num} Materials Comparison", fontsize=20, weight='bold')

    plt.tight_layout()

    plt.show()

*def* main():

    print("Material Analysis System")

    print("-----------------------")

    print("1. Case 1")

    print("2. Case 2")

    while True:

        case = input("\nSelect case to analyze (1 or 2): ")

        if case in ['1', '2']:

            case = *int*(case)

            break

        print("Invalid input. Please enter 1 or 2.")

    w_m, w_t, w_c = determine_weights()

    if case == 1:

        materiales = {

            "CFRP": {

                "Yield strength (MPa)": 800,

                "Fatigue strength (MPa)": 225,

                "Young modulus (MPa)": 114000,

                "Max service temperature (°C)": 220,

                "Min service temperature (°C)": -123,

                "Flammability": 2,

                "Resistance to water": 4,

                "Resistance to acids": 2,

                "Resistance to alkalis": 3,

                "Resistance to fuel, oils, solvents": 3,

                "Resistance to alcohols, aldehydes, ketones": 2,

                "Resistance to UV Radiation": 3,

                "Footprint - Primary Production": 50.5,

                "CO2 Combustion": 3.33,

                "CO2 Processing": 1.08,

            },

            "Cast Iron": {

                "Yield strength (MPa)": 438,

                "Fatigue strength (MPa)": 256,

                "Young modulus (MPa)": 175000,

                "Max service temperature (°C)": 450,

                "Min service temperature (°C)": -69.2,

                "Flammability": 4,

                "Resistance to water": 3,

                "Resistance to acids": 2,

                "Resistance to alkalis": 3,

                "Resistance to fuel, oils, solvents": 4,

                "Resistance to alcohols, aldehydes, ketones": 3,

                "Resistance to UV Radiation": 4,

                "Footprint - Primary Production": 2.49,

                "CO2 Combustion": 0.703,

                "CO2 Processing": 0.865,

            },

            "GFRP": {

                "Yield strength (MPa)": 255.5,

                "Fatigue strength (MPa)": 66.2,

                "Young modulus (MPa)": 21400,

                "Max service temperature (°C)": 220,

                "Min service temperature (°C)": -123,

                "Flammability": 2,

                "Resistance to water": 4,

                "Resistance to acids": 2,

                "Resistance to alkalis": 3,

                "Resistance to fuel, oils, solvents": 3,

                "Resistance to alcohols, aldehydes, ketones": 2,

                "Resistance to UV Radiation": 2,

                "Footprint - Primary Production": 6.32,

                "CO2 Combustion": 1.02,

                "CO2 Processing": 1.08,

            },

            "PE": {

                "Yield strength (MPa)": 23.45,

                "Fatigue strength (MPa)": 22,

                "Young modulus (MPa)": 758.5,

                "Max service temperature (°C)": 110,

                "Min service temperature (°C)": -123,

                "Flammability": 1,

                "Resistance to water": 4,

                "Resistance to acids": 4,

                "Resistance to alkalis": 4,

                "Resistance to fuel, oils, solvents": 3,

                "Resistance to alcohols, aldehydes, ketones": 4,

                "Resistance to UV Radiation": 2,

                "Footprint - Primary Production": 1.95,

                "CO2 Combustion": 0.99,

                "CO2 Processing": 1.73,

            },

            "Steel": {

                "Yield strength (MPa)": 652.5,

                "Fatigue strength (MPa)": 389,

                "Young modulus (MPa)": 210000,

                "Max service temperature (°C)": 350,

                "Min service temperature (°C)": -53.2,

                "Flammability": 4,

                "Resistance to water": 3,

                "Resistance to acids": 1,

                "Resistance to alkalis": 3,

                "Resistance to fuel, oils, solvents": 4,

                "Resistance to alcohols, aldehydes, ketones": 3,

                "Resistance to UV Radiation": 4,

                "Footprint - Primary Production": 2.49,

                "CO2 Combustion": 0.703,

                "CO2 Processing": 0.897,

            },

            "Concrete": {

                "Yield strength (MPa)": 2,

                "Fatigue strength (MPa)": 1.15,

                "Young modulus (MPa)": 35500,

                "Max service temperature (°C)": 977,

                "Min service temperature (°C)": -160,

                "Flammability": 4,

                "Resistance to water": 4,

                "Resistance to acids": 2,

                "Resistance to alkalis": 3,

                "Resistance to fuel, oils, solvents": 3,

                "Resistance to alcohols, aldehydes, ketones":2,

                "Resistance to UV Radiation": 4,

                "Footprint - Primary Production": 0.128,

                "CO2 Combustion": 0.24,

                "CO2 Processing": 0.44,

            },

            "Aluminum": {

                "Yield strength (MPa)": 127.5,

                "Fatigue strength (MPa)": 98.6,

                "Young modulus (MPa)": 68300,

                "Max service temperature (°C)": 150,

                "Min service temperature (°C)": -273,

                "Flammability": 4,

                "Resistance to water": 4,

                "Resistance to acids": 4,

                "Resistance to alkalis": 2,

                "Resistance to fuel, oils, solvents": 4,

                "Resistance to alcohols, aldehydes, ketones":3,

                "Resistance to UV Radiation": 4,

                "Footprint - Primary Production": 13.9,

                "CO2 Combustion": 2.81,

                "CO2 Processing": 0.375,

            },

            "Teak": {

                "Yield strength (MPa)": 56.7,

                "Fatigue strength (MPa)": 30.25,

                "Young modulus (MPa)": 11750,

                "Max service temperature (°C)": 150,

                "Min service temperature (°C)": -273,

                "Flammability": 4,

                "Resistance to water": 4,

                "Resistance to acids": 4,

                "Resistance to alkalis": 1,

                "Resistance to fuel, oils, solvents": 4,

                "Resistance to alcohols, aldehydes, ketones": 3,

                "Resistance to UV Radiation": 4,

                "Footprint - Primary Production": 0.578,

                "CO2 Combustion": 1.78,

                "CO2 Processing": 1.38,

            }

        }

        calcular_x = True

    else:

        materiales = {

            "ABS": {

                "Yield strength (MPa)": 42.05,

                "Fatigue strength (MPa)": 17.95,

                "Young modulus (MPa)": 2415,

                "Max service temperature (°C)": 76.9,

                "Min service temperature (°C)": -45.2,

                "Flammability": 1,

                "Resistance to water": 4,

                "Resistance to acids": 3,

                "Resistance to alkalis": 4,

                "Resistance to fuel, oils, solvents": 2,

                "Resistance to alcohols, aldehydes, ketones": 2,

                "Resistance to UV Radiation": 1,

                "Footprint - Primary Production": 3.77,

                "CO2 Combustion": 1.63,

                "CO2 Processing": 1.32,

            },

            "Polyamides (PA)": {

                "Yield strength (MPa)": 46.5,

                "Fatigue strength (MPa)": 21,

                "Young modulus (MPa)": 1490,

                "Max service temperature (°C)": 130,

                "Min service temperature (°C)": -80,

                "Flammability": 2,

                "Resistance to water": 3,

                "Resistance to acids": 1,

                "Resistance to alkalis": 1,

                "Resistance to fuel, oils, solvents": 4,

                "Resistance to alcohols, aldehydes, ketones": 2,

                "Resistance to UV Radiation": 2,

                "Footprint - Primary Production": 8.0,

                "CO2 Combustion": 1.72,

                "CO2 Processing": 3.19,

            },

            "Polylactide (PLA)": {

                "Yield strength (MPa)": 52.5,

                "Fatigue strength (MPa)": 24.95,

                "Young modulus (MPa)": 3450,

                "Max service temperature (°C)": 54.9,

                "Min service temperature (°C)": -20.2,

                "Flammability": 2,

                "Resistance to water": 3,

                "Resistance to acids": 1,

                "Resistance to alkalis": 1,

                "Resistance to fuel, oils, solvents": 2,

                "Resistance to alcohols, aldehydes, ketones": 1,

                "Resistance to UV Radiation": 3,

                "Footprint - Primary Production": 2.4,

                "CO2 Combustion": 1.1,

                "CO2 Processing": 0.816,

            },

            "Polypropylene (PP)": {

                "Yield strength (MPa)": 26.25,

                "Fatigue strength (MPa)": 7.83,

                "Young modulus (MPa)": 922,

                "Max service temperature (°C)": 83.9,

                "Min service temperature (°C)": -25.2,

                "Flammability": 1,

                "Resistance to water": 4,

                "Resistance to acids": 3,

                "Resistance to alkalis": 4,

                "Resistance to fuel, oils, solvents": 2,

                "Resistance to alcohols, aldehydes, ketones": 2,

                "Resistance to UV Radiation": 1,

                "Footprint - Primary Production": 3.01,

                "CO2 Combustion": 1.0,

                "CO2 Processing": 1.66,

            },

            "Polycarbonate (PC)": {

                "Yield strength (MPa)": 62.1,

                "Fatigue strength (MPa)": 27.25,

                "Young modulus (MPa)": 2380,

                "Max service temperature (°C)": 116,

                "Min service temperature (°C)": -47.2,

                "Flammability": 2,

                "Resistance to water": 4,

                "Resistance to acids": 4,

                "Resistance to alkalis": 3,

                "Resistance to fuel, oils, solvents": 3,

                "Resistance to alcohols, aldehydes, ketones": 3,

                "Resistance to UV Radiation": 2,

                "Footprint - Primary Production": 6.18,

                "CO2 Combustion": 2.11,

                "CO2 Processing": 1.46,

            },

            "Material X": {

                "Yield strength (MPa)": 75,

                "Fatigue strength (MPa)": 35,

                "Young modulus (MPa)": 3500,

                "Max service temperature (°C)": 260,

                "Min service temperature (°C)": -200,

                "Flammability": 3,

                "Resistance to water": 4,

                "Resistance to acids": 4,

                "Resistance to alkalis": 4,

                "Resistance to fuel, oils, solvents": 4,

                "Resistance to alcohols, aldehydes, ketones": 4,

                "Resistance to UV Radiation": 3,

                "Footprint - Primary Production": 0.94,

                "CO2 Combustion": 1.1,

                "CO2 Processing": 0.32,

            }

        }

        calcular_x = False

    df_final, materiales = calcular_parametros(materiales, case, w_m, w_t, w_c, calcular_x)

    if input("\nShow detailed material properties? (y/n): ").lower() == 'y':

        mostrar_propiedades(materiales, case)

    print("\nAnalysis completed.")

    mostrar_resultados(df_final, case)

    generar_grafica_polar(df_final, case)

if __name__ == "__main__":

    main()
